# 4,5,7‐Trisubstituted indeno[1,2‐*b*]indole inhibits CK2 activity in tumor cells equivalent to CX‐4945 and shows strong anti‐migratory effects

**DOI:** 10.1002/2211-5463.13346

**Published:** 2021-12-18

**Authors:** Robin Birus, Ehab El‐Awaad, Laurens Ballentin, Faten Alchab, Dagmar Aichele, Laurent Ettouati, Claudia Götz, Marc Le Borgne, Joachim Jose

**Affiliations:** ^1^ Institute of Pharmaceutical and Medicinal Chemistry Westfälische Wilhelms‐Universtität Münster Germany; ^2^ Department of Pharmacology Faculty of Medicine Assiut University Egypt; ^3^ EEA 4446 Bioactive Molecules and Medicinal Chemistry, Faculté de Pharmacie‐ISPB, SFR Santé Lyon‐Est CNRS UMS3453‐INSERM US7 Université Claude Bernard Lyon 1, Université de Lyon France; ^4^ Faculty of Pharmacy Manara University Latakia Syria; ^5^ CNRS UMR 5246 Institut de Chimie et Biochimie Moléculaires et Supramoléculaires (ICBMS), Faculté de Pharmacie, ISPB Université Lyon 1, Université de Lyon France; ^6^ Medical Biochemistry and Molecular Biology Saarland University Germany; ^7^ Small Molecules for Biological Targets Team, Centre de recherche en cancérologie de Lyon, Centre Léon Bérard, CNRS 5286, INSERM 1052 Université Claude Bernard Lyon 1, Université de Lyon France

**Keywords:** CK2, HPLC‐MS/MS, indeno[1,2‐*b*]indole, live‐cell imaging, pharmacokinetics

## Abstract

Highly pleiotropic and constitutively active protein kinase CK2 is a key target in cancer therapy, but only one small‐molecule inhibitor has reached clinical trials—CX‐4945. In this study, we present the indeno[1,2‐*b*]indole derivative 5‐isopropyl‐4‐methoxy‐7‐methyl‐5,6,7,8‐tetrahydroindeno[1,2‐*b*]indole‐9,10‐dione (**5a‐2**) that decreased the intracellular CK2 activity in A431, A549, and LNCaP tumor cell lines analogous to CX‐4945 (> 75% inhibition at 20 µm) and similarly blocked CK2‐specific Akt phosphorylation in LNCaP cells. Cellular uptake analysis demonstrated higher intracellular concentrations of **5a‐2** (408.3 nm) compared with CX‐4945 (119.3 nm). This finding clarifies the comparable effects of both compounds on the intracellular CK2 activity despite their different inhibitory potency *in vitro* [IC_50_ = 25 nm (**5a‐2**) and 3.7 nm (CX‐4945)]. Examination of the effects of both CK2 inhibitors on cancer cells using live‐cell imaging revealed notable differences. Whereas CX‐4945 showed a stronger pro‐apoptotic effect on tumor cells, **5a‐2** was more effective in inhibiting tumor cell migration. Our results showed that 49% of intracellular CX‐4945 was localized in the nuclear fraction, whereas 71% of **5a‐2** was detectable in the cytoplasm. The different subcellular distribution, and thus the site of CK2 inhibition, provides a possible explanation for the different cellular effects. Our study indicates that investigating CK2 inhibition‐mediated cellular effects in relation to the subcellular sites of CK2 inhibition may help to improve our understanding of the preferential roles of CK2 within different cancer cell compartments.

## Graphical abstract

In this study, a 4,5,7‐trisubstituted indeno[1,2‐*b*]indole, **5a‐2**, is shown to inhibit CK2 activity in cancer cells equivalently to the well‐known inhibitor CX‐4945. Whereas CX‐4945 shows strong pro‐apoptotic and anti‐proliferative effects, we demonstrate a strong anti‐migratory effect for **5a‐2**. We conclude that subcellular localization might be critical for the differential cellular effects of CK2 inhibitors.

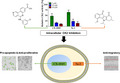

**Abbreviations**
AKT: protein kinase B
CE: capillary electrophoresis
HPLC: high‐performance liquid chromatography
LIF: laser‐induced fluorescence
MOPS: 3‐(N‐morpholino)propane sulfonic acid
MS: mass spectrometry
SAR: structure–activity relationship

Human protein kinase CK2 is a constitutively active serine/threonine kinase [1]. Today, the enzyme, which was first described in 1954 as ‘casein kinase 2’, is known to phosphorylate more than 500 substrates [2, 3]. Together with an ubiquitous expression in eukaryotic cells [4], this high pleiotropy is the reason for an involvement of CK2 in numerous cellular processes [5, 6]. These include cell movement, proliferation, and apoptosis [7, 8, 9]. The subcellular localization of protein kinase CK2 is known to be tightly regulated as different protein substrates and functions of CK2 are found in different cellular compartments [4]. As CK2 is dynamically distributed within various cell compartments, it is thought that CK2 activity can be regulated based on its site of action in the cell [10, 11].

CK2 is a well‐established therapeutic target in cancer [12, 13, 14, 15]. The activity of the protein kinase was shown to be upregulated in various types of cancer cells being essential for their survival. Inhibition of CK2 activity in turn leads to the induction of apoptosis in cancer cells [12, 14, 16, 17, 18, 19]. In contrast, non‐cancer cells are less sensitive to inhibition of the protein kinase under analogous conditions [20]. Hence, CK2 inhibition is believed to be an effective strategy in tumor therapy. An intensified search for selective CK2 inhibitors has been carried out in recent decades [21, 22]. In addition to the peptide‐based CK2 inhibitor CIGB‐300 [23, 24], one small‐molecule CK2 inhibitor named silmitasertib (CX‐4945) is being investigated in clinical trials. This inhibitor has proven its strong anti‐cancer effects in several preclinical [25, 26, 27, 28] and clinical studies [29, 30, 31]. Several investigations have focused on the cellular effects of CX‐4945 that could be attributed to inhibition of CK2 [32, 33, 34]. However, less is known about the subcellular distribution of the compound, which would reflect the level of CK2 inhibition within different cell compartments.

Throughout the search for lead structures to inhibit CK2, indeno[1,2‐b]indoles were identified as potent, ATP‐competitive compounds. Their flat and tetracyclic structure designed to address the ATP‐binding pocket of CK2 allows a diverse derivatization of the ring system. Therefore, numerous indeno[1,2‐*b*]indole derivatives inhibiting this protein kinase have already been described [35, 36, 37]. One of the most potent indeno[1,2‐*b*]indoles as CK2 inhibitor is the compound 4p (5‐isopropyl‐4‐(3‐methylbut‐2‐enyloxy)‐5,6,7,8‐tetrahydroindeno[1,2‐*b*]indole‐9,10‐dione), which inhibited the activity of the protein kinase *in vitro* with an IC_50_ value of 25 nm. Furthermore, 4p was shown to inhibit intracellular CK2 activity and to exhibit anti‐cancer effects on breast, prostate, skin, and lung cancer cells [38, 39]. Detailed structure–activity relationship (SAR) studies showed that an *N*‐isopropyl group in position 5 of the indeno[1,2‐*b*]indole scaffold, as well as an alkyl group in position 7 and an alkoxy group in position 4, increased the inhibitory potential of the compounds [36, 38].

Based on our knowledge from the SAR analysis, we developed new 4,5,7‐trisubstituted indeno[1,2‐*b*]indole derivatives, which showed strong inhibitory effects against protein kinase CK2 *in vitro*. Using CX‐4945 as a reference compound, the most potent CK2 inhibitor **5a‐2** was examined for its effects on intracellular CK2 activity in three different tumor cell lines. In addition, the cellular uptake of **5a‐2** was analyzed to estimate the amount of the compound that could interact with the target in the cell. Subsequently, we examined the changes in cell proliferation, apoptosis, and migration mediated by this indeno[1,2‐*b*]indole as compared to those induced by CX‐4945. To help clarify the observed preferential anti‐cancer effects for the investigated compound, we compared its subcellular distribution with that of CX‐4945. Through linking the cellular effects mediated by CK2 inhibition with subcellular distribution of the investigated compounds, this study helps to provide hints for the impact of differential CK2 inhibition in cell compartments on several biological processes.

## Materials and methods

### Chemical synthesis

All chemicals were purchased in the highest purity available. The four tetrahydroindeno[1,2‐*b*]indole‐9,10‐diones **5a,b** were synthesized according to previously reported methods [38, 40, 41] (detailed description of the synthetic pathway can be found in Supporting Information; Scheme S1). Compounds **5a,b** and CX‐4945 were dissolved at a concentration of 10 mm in dimethyl sulfoxide (DMSO). Stock solutions were stored at −20 °C. Microwave reactions were done on a Biotage Initiator Microwave synthesizer 2.0 440 W. Melting points were determined on an Electrothermal 9200 capillary apparatus. The IR spectra were recorded on a Perkin Elmer Spectrum Two IR Spectrometer. The ^1^H and ^13^C NMR spectra (Figs. S1–S8) were recorded at 400 MHz on a Brücker DRX 400 spectrometer. Chemical shifts are expressed in ppm (δ) downfield from internal tetramethylsilane and coupling constants *J* are reported in hertz (Hz) (Figs. S1–S8). The mass spectra were performed by direct ionization (EI or CI) on a ThermoFinnigan MAT 95 XL apparatus. Chromatographic separations were performed on silica gel columns by column chromatography (Kieselgel 300–400 mesh). All reactions were monitored by TLC on GF254 plates that were visualized under a UV lamp (254 nm). Evaporation of solvent was performed in vacuum with rotating evaporator. The purity of the final compounds (> 95%) was determined by uHPLC/MS on an Agilent 1290 system using a Agilent 1290 Infinity ZORBAX Eclipse Plus C18 column (2.1 x 50 mm, 1.8 µm particle size) with a gradient mobile phase of H_2_O/CH_3_CN (90 : 10, v/v) and 0.1% of formic acid to H_2_O/CH_3_CN (10 : 90, v/v) and 0.1% of formic acid at a flow rate of 0.5 mL·min^−1^. UV monitoring at the wavelength of 254 nm with a runtime of 10 min was performed.

### *In vitro* IC_50_ determination

IC_50_ values of the CK2 inhibitors were determined using capillary electrophoresis‐based (CE) assay established by Gratz et al. [42]. CK2 inhibitors were added in an amount of 2 µL (stock solution in DMSO) to 78 µL kinase buffer (50 mm Tris/HCl pH 7.5, 100 mm NaCl, 10 mm MgCl_2_) containing 0.25 µg CK2 holoenzyme. The solutions were preincubated at 37 °C for 10 min. Afterward, 120 µL assay buffer (25 mm Tris/HCl pH 8.5, 150 mm NaCl, 5 mm MgCl_2_, 190 µm of the substrate peptide RRRDDDSDDD, 100 µm ATP) were added. After incubation at 37 °C for 15 min, the reaction was stopped by transferring the samples on ice and adding 5 mm EDTA. The samples were investigated via CE. We analyzed each inhibitor in nine different concentrations. As control for 0% inhibition, DMSO without inhibitor was added to a sample. Samples without ATP served as control for 100% inhibition. IC_50_ values were calculated from the corresponding dose‐response curves via graphpad Prism 5 (graphpad Software, La Jolla, CA, USA).

### LogD_7.4_ determination

The determination of the LogD_7.4_ values was performed using a shake flask method developed by Galla et al. 2016 [43]. Briefly, LogD_7.4_ values of the CK2 inhibitors were determined in three preparations with different *n*‐octanol/MOPS (3‐(N‐morpholino)propanesulfonic acid) buffer ratios (2 : 1; 1 : 1; 1 : 2). After the MOPS buffer phase was adjusted to pH 7.4, 1 µm of the corresponding CK2 inhibitor was dissolved in it. MOPS buffer and *n*‐Octanol phases were transferred into microreaction tubes in their respective ratios. Afterward, samples were vortexed for 2 min. Subsequently, the samples were centrifuged for 10 min at 13,000 × ***g***. Inhibitor concentrations were determined in the MOPS buffer phase by HPLC‐MS/MS. LogD_7.4_ were calculated as indicated in Equation 1.

(1) $$\text{Log}D_{7.4}=\log\frac{c_{n‐\text{octanol}}}{c_{\text{buffer}}}=\log\frac{c_{\text{total}}‐c_{\text{buffer}}}{c_{\text{buffer}}}$$

whereby c*_n_*_‐octanol_ is the inhibitor concentration within the *n*‐octanol phase and c_buffer_ the concentration within the MOPS buffer phase.

### Cell lines

Human epidermoid carcinoma cells A431 and human lung cancer cells A549, kindly provided by the Department of Experimental Tumor Biology, University of Münster, were cultured in DMEM high glucose medium (Life Technologies, Massachusetts, MA, USA) supplemented with 2 mm l‐glutamine and 10% fetal calf serum (FCS, Life Technologies, Massachusetts, MA, USA). Lymph node‐metastasized prostate carcinoma cells LNCaP, kindly provided by the Department of Medical Biochemistry and Molecular Biology, Saarland University, were cultured in RPMI 1640 medium GlutaMax (Life Technologies, Massachusetts, MA, USA) supplemented with 10% FCS. Human umbilical vein endothelial cells (HUVEC), prepared and provided by the Center for Molecular Biology of Inflammation, University of Münster, were cultured in endothelial growth medium 2 (Promocell, Heidelberg, Germany) supplemented with 30 µg·mL^−1^ gentamicin and 15 ng·mL^−1^ amphotericin B (Merck Millipore, Darmstadt, Germany). Cells were seeded at a density of 5.0 × 10^5^ cells per well in 6‐well and at 1.0 × 10^4^ in 96‐well plates (Greiner, Darmstadt, Germany).

### Immunocytochemical CK2α detection

CK2α expression pattern was analyzed in HUVECs, A431, A549, and LNCaP cells. For this purpose, cells were cultured on coverslips in 6‐well plates coated with 0.1 mg·mL^−1^ poly‐l‐lysine (Sigma‐Aldrich, Steinheim, Germany). Cells were cultured to a confluence of 50 – 70%, washed with PBS, and fixed with 4% paraformaldehyde (PFA, Merck Millipore, Darmstadt, Germany) for 10 min subsequently. Cells were permeabilized with 0.1% Triton X‐100 (AppliChem, Darmstadt, Germany) for 10 min, before blocking with 3% bovine serum albumin (BSA, Roth, Karlsruhe, Germany) for 1 h. After that, cells were incubated with a CK2α‐specific antibody (1AD9, Santa Cruz Biotechnology, Santa Cruz, CA, USA) or vehicle control at 4 °C overnight. After washing with PBS, cells were incubated with a corresponding secondary antibody labeled with the fluorophore DyLight® 488 (35502, Thermo Scientific, Braunschweig, Germany) for 1 h in the dark. The secondary antibody solution also contained a DNA‐binding fluorescent dye for cell nuclei staining (Hoechst 33342, Sigma‐Aldrich, Steinheim, Germany). After washing with PBS, coverslips were preserved on microscopy slides. The preparations were examined by fluorescence microscopy (BZ‐9000, Keyence, Osaka, Japan) obtained with a 100‐fold lens. For comparing the level of fluorescence in the nuclear region between the different cell lines, corrected total cellular fluorescence (CTCF) was determined in the nuclear and cytoplasmic regions using imagej software (imagej 1.50i, NIH, USA) according to the equation:

CTCF = integrated density – (area of selected region of interest × mean fluorescence of background readings) [44].

Mean ratios of nuclear/cytoplasmic CTCF values were determined for randomly selected stained cells from each cell line and plotted using graphpad Prism 5 (graphpad Software, La Jolla, CA, USA).

### Analysis of Akt phosphorylation

For Akt phosphorylation analysis, LNCaP cells were cultured in 6‐well plates for 48 h. Afterward, cells were treated with either vehicle control (1% DMSO) or **5a‐2**, **5b‐2,** or CX‐4945 at 20 µm for 24 h. The cells were detached from the culture plates using cell scrapers, sedimented by centrifugation at 700 × ***g*** and 4 °C for 5 min, washed with PBS, and lysed by suspending in 100 µL homogenization buffer (50 mm Tris/HCl (pH 7.5), 1 mm EDTA, 1 mm Na_3_VO_4_, 0.5 mm NaF, 0.1 mm PMSF, 1 mm benzamidine, and 1% Triton X‐100). Akt and pAkt^S129^ were analyzed in the cell lysates by immunoblotting as described by Schmitt et al. [45] using the following antibodies:

Polyclonal anti‐Akt antibody (#9272, Cell Signaling Technology, Danvers, MA, USA), monoclonal anti‐Akt1 (phospho S129) antibody [EPR6150] (#ab133458, Abcam, Cambridge, UK), and anti‐CK2α‐specific antiserum (kindly provided by the Department of Medical Biochemistry and Molecular Biology, Saarland University, #26 as described by Faust et al. [46]). α‐Tubulin served as a loading control (anti‐α‐tubulin, monoclonal, #66031, Proteintech, St. Leon‐Rot, Germany). A protein amount of 60 µg was analyzed per sample.

### Determination of intracellular CK2 activity

To analyze the influence of CK2 inhibitors on the intracellular CK2 activity, an adapted experimental procedure described by Schneider et al. [47] combined with a capillary electrophoresis (CE) developed by our group [39] was used. A431, A549, and LNCaP cells were cultured in 6‐well cell plates for 48 h, before treatment with either vehicle control (1% DMSO) or CK2 inhibitors at 1, 10, and 20 µm for 24 h. The cells were detached from the cell culture plates with cell scrapers, sedimented via centrifugation at 700 × ***g*** and 4 °C for 5 min, and washed with PBS. Afterward, cells were lysed by suspending in 100 µL lysis buffer (50 mm Tris/HCl (pH 7.5), 0.15 mm NaCl, 1 mm Na_3_VO_4_, 0.5 mm NaF, 0.8 µm aprotinin, 0.01 mm pepstatin A, 0.02 mm leupeptin 0.1 mm PMSF, 1 mm benzamidine, 0.5% sodium deoxycholate, and 1% Triton X‐100). Cell debris was removed by centrifugation at 10,000 × ***g*** and 4 °C for 10 min. Protein concentrations were determined via Bradford analysis (Roti‐Quant, Roth, Karlsruhe, Germany). 80 µL kinase buffer (50 mm Tris/HCl pH 7.5, 100 mm NaCl, 10 mm MgCl_2_) containing 90 µg protein per sample was preincubated at 37 °C for 10 min. Afterward, 120 µL assay buffer (25 mm Tris/HCl pH 8.5, 150 mm NaCl, 5 mm MgCl_2_, 190 µm FITC‐RRRDDDSDDD‐NH_2_, 100 µm ATP) were added. The samples were incubated at 37 °C for 15 min. The reaction was stopped by transferring the samples on ice and adding 5 mm EDTA. Intracellular CK2 activities were determined via CE analysis.

### Cellular uptake determination

The cellular uptake of CK2 inhibitors was investigated representative in A431 cells. Cells were cultured for 48 h at 37 °C and 5% CO_2_ in 6‐well plates. Afterward, cells were treated with either 1 or 3 µm inhibitors for 1, 5, and 12 h. Cells were washed with PBS twice and detached using trypsin/EDTA. The number of cells in each sample was determined with an automated cell counter (Scepter®, Merck Millipore, Darmstadt, Germany). After centrifugation at 700 × ***g*** and 4 °C for 5 min, cells were washed multiple times with PBS followed by lysis in 100 µL homogenization buffer (50 mm Tris/HCl (pH 7.5), 1 mm EDTA, 1 mm Na_3_VO_4_, 0.5 mm NaF, 0.1 mm PMSF, 1 mm benzamidine, and 1% Triton X‐100). Proteins were precipitated by adding 500 µL ice‐cold ACN/MeOH 1/1 (v/v). Samples were centrifuged 30 min at 20,000 × ***g*** and 4 °C. Supernatants were evaporated to dryness using a vacuum centrifuge maintained at 50 °C under full vacuum for 2 h. Residues were resolved in 100 µL ACN/H_2_O 1/1 (v/v) and analyzed via HPLC‐MS/MS. Intracellular inhibitor concentrations were calculated according to Rahnel et al. [48] as indicated in Equation 2.

(2) $$c_{x}=\frac{A_{x}‐b}{a}\times V_{\text{final}}\times \frac{1}{N_{\text{cell}}\times \frac{4}{3}\pi \times {\left(\frac{d_{\text{cell}}}{2\times 1000}\right)}^{3}}$$

For the equation above, c_x_ is the intracellular inhibitor concentration, *A*_x_ the peak area of the analyte, b is the intercept and a the slope of the external calibration curve, *V*_final_ is the final sample volume, *N*_cell_ is the number of cells, and *d*_cell_ is the mean diameter of the cells.

### Temperature‐dependent uptake analysis

To investigate the temperature dependency of the cellular uptake of CK2 inhibitors, cells were treated as described above. After treatment with CK2 inhibitors at 1 µm, cells were incubated at either 37 °C or 4 °C under atmospheric conditions for 3 h. Afterward, cell lysis and inhibitor quantification were conducted analogous to the previous chapter.

### EC_50_ determination

For the determination of EC_50_ values, A431, A549, LNCaP, and HUVEC cells were cultured in 96‐well plates. The cell confluence in each well was determined using IncuCyte® S3 live‐cell imaging system (Sartorius, Michigan, MI, USA). After the cell confluence reached 30%, cells were treated with inhibitors at eight different concentrations between 0.1 and 50 µm or vehicle control (1% DMSO). The cell confluence was monitored in 2 h intervals for a total period of 48 h. Confluence values were plotted against time points resulting in growth curves of the tumor cells for each inhibitor concentration. AUCs of all growth curves were calculated using graphpad Prism 5 (graphpad Software, La Jolla, CA, USA) and normalized to the AUC of the vehicle control. These relative AUCs were plotted against the logarithmic inhibitor concentration. Using graphpad Prism 5, EC_50_ values were determined from a nonlinear regression.

### Investigation of apoptosis

To investigate the induction of tumor cell apoptosis, A431 cells were cultured in 96‐well cell culture plates to a confluence of 30% overnight. Afterward, the cells were treated with either vehicle control (1% DMSO) or CK2 inhibitors at 1, 10, and 20 µm. Each well received IncuCyte® caspase 3/7 green reagent (5 µm final concentration, Sartorius, Michigan, MI, USA) as well. The apoptotic cell count (1/well) depicted from the green fluorescent signals was monitored for 48 h using IncuCyte® S3 live‐cell imaging system. The apoptotic cell counts in control wells were subtracted as background from those in treated wells.

### Tumor cell migration analysis

IncuCyte® 96‐Well ImageLock plates (Sartorius, Michigan, MI, USA) were coated with 300 µg·mL^−1^ rat coil Collagen‐I in 20 mm acetic acid. After seeding in the coated plates, A549 cells were cultured to a confluence of 100%. Scratch wounds were created in all wells using IncuCyte® 96‐well WoundMaker tool (Sartorius, Michigan, MI, USA). Relative wound density (%) of cells treated with vehicle control (1% DMSO) or the selected CK2 inhibitors at 1 and 10 µm was monitored for 48 h using IncuCyte® S3 live‐cell imaging system.

### Subcellular distribution analysis

LNCaP cells were used to analyze the subcellular distribution of CK2 inhibitors. Cells were cultured in 75‐cm^2^ culture flasks (Greiner, Darmstadt, Germany) to a confluence of approximately 90%. Afterward, cells were treated with the corresponding CK2 inhibitor at 1 µm for 5 h. Then, cells were harvested using cell scrapers, washed with PBS, suspended in 1 mL fractionation buffer (20 mm HEPES, 10 mm KCl, 2 mm MgCl_2_, 1 mm EDTA, 1 mm EGTA, 1 mm DTT, 1 mm Na_3_VO_4_, 1 mm benzamidine, 0.5 mm NaF, and 0.1 mm PMSF) and lysed mechanically. Subcellular fractions were separated and collected according to an Abcam protocol (https://www.abcam.com/protocols/subcellular‐fractionation‐protocol). After separation, each fraction was suspended in 200 µL 1% Triton X‐100 in PBS. Proteins were precipitated by adding 500 µL ice‐cold ACN/MeOH 1/1 (v/v) and sedimented via centrifugation for 30 min at 4 °C and 20,000 × ***g***. Supernatants were evaporated to dryness using a vacuum centrifuge maintained at 50 °C under full vacuum for 2 h. Residues were solved in 200 µL ACN/H_2_O 1/1 (v/v). CK2 inhibitors were detected in each fraction using HPLC‐MS/MS. AUCs of the analyte peaks in each fraction were summed and related to each other according to equation 3.

(3) $$\text{Relative amount}\;\left[\%\right]=\frac{A_{x}}{\sum A_{\text{xi - iv}}}\times 100\%$$

Here, A_x_ is the area of the signal of the CK2 inhibitor in the respective fraction and ∑*A*_xi‐iv_ is the sum of the areas of the signals of all four cellular fractions examined.

### Capillary electrophoresis analysis of kinase reaction products

Capillary electrophoresis (CE) analysis was conducted as previously described by our group [39, 42]. Therefore, we used a PA800 plus capillary electrophoresis system (AB Sciex, Darmstadt, Germany). A bare fused silica capillary (BGB, Rheinfelden, Germany) was used for sample separation. Analyte detection was performed via either diode array detection at 204 nm or laser‐induced fluorescence detection (excitation: 488 nm; emission: 518 nm). Acetic acid (2 m, adjusted to pH 1.8 using conc. HCl) served as background electrolyte.

### HPLC‐MS/MS analysis

For determination of LogD_7.4_ values, cellular uptake, and subcellular distribution, a Nexera X2 HPLC system (Shimadzu, Kyoto, Japan) was used. The system was combined with an XSelect HSS T3 analytical column (100 mm × 2.10 mm, 2.5 µm, Waters, Massachusetts, MA, USA), maintained at 40 °C. A gradient solvent flow (A: ACN/H_2_O 10/90 + 0.1% formic acid; B: ACN/H2O 90/10 + 0.1% formic acid) was run with a flow rate of 0.3 mL·min^−1^ and adjusted for analyte separation as follows: From 0% B to 100% B: 0 – 7 min; 100% B: 7 – 9 min; from 100% B to 0%: 9 – 9.5 min; 0% B: 9.5 – 15 min. The injection volume was 5 µL. Analytes were detected with a QTrap® 6500^+^ mass spectrometer (AB Sciex, Darmstadt, Germany), which was run in MRM mode. The ion source was operated in ESI mode. MS parameters were optimized for the corresponding analyte in each case. Quantification was conducted using an external calibration.

### Statistical analysis

The mean values for corrected total cellular fluorescence (CTCF) ratios of the three tumor cell lines were compared to that of HUVEC cells, and the EC_50_ values of **5a‐2** on three different tumor cell lines were compared to those of CX‐4945 using a two‐tailed unpaired Student’s *t*‐test (GRAPHPAD Prism 5). The *P*‐values are given as follows: *P < 0.05, **P < 0.01.

## Results

### Inhibition of CK2 *in vitro*

**Fig. 1:**
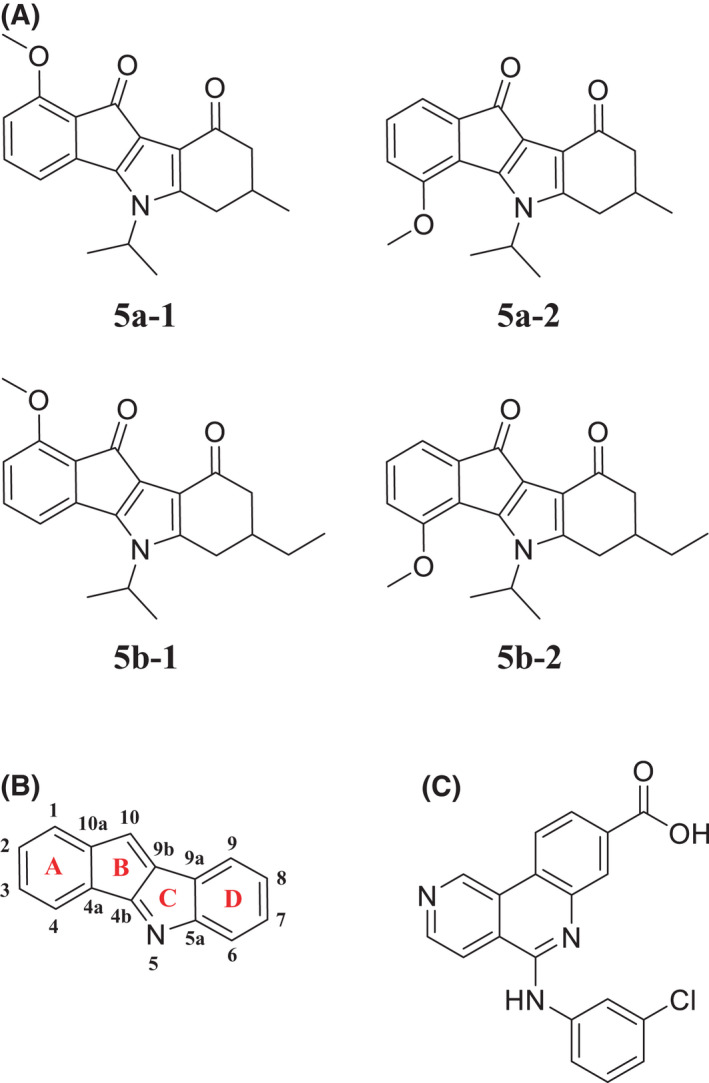
Structures of CK2 inhibitors that were investigated in this study. (A) 4,5,7‐Trisubstituted indeno[1,2‐*b*]indole derivatives investigated here (B) General indeno[1,2‐*b*]indole scaffold with numbered carbon atoms. (C) CX‐4945 (silmitasertib) served as a reference.

The ability of four 4,5,7‐trisubstituted indeno[1,2‐*b*]indoles (Fig. 1A) to inhibit the kinase activity of CK2 *in vitro* was determined by a capillary electrophoretic method as previously described [42]. Compounds **5a‐1** and **5b‐1** exhibited IC_50_ values of 8170 nm and 9910 nm (Table 1), respectively, while the IC_50_ values of their regioisomers **5a‐2** and **5b‐2** were drastically lower (25 nm and 47 nm, respectively). Thus, the latter two compounds were 200‐ to 350‐fold more potent with respect to inhibition of CK2 activity *in vitro*. These results indicate that a methoxy group in position 4 of the indeno[1,2‐*b*]indole scaffold (Fig. 1B) is more favorable for CK2 inhibition than in position 1. Compounds **5a‐1** and **5b‐1** were not included in further investigations because of their weak inhibitory effects compared to their regioisomers **5a‐2** and **5b‐2**. Notably, the alkyl substituent in position 7 seems to influence the inhibitory effect of indeno[1,2‐*b*]indoles on CK2 activity as well. Compound **5a‐2**, with a methyl group in position 7, exhibited a 1.9‐fold lower IC_50_ compared to compound **5b‐2** that has an ethyl group in the same position. Compared to the reference CK2 inhibitor CX‐4945 (Fig. 1C), both **5a‐2** and **5b‐2** were, however, less potent *in vitro* since CX‐4945 had an IC_50_ value of 3.7 nm using the same CK2 activity assay [38].

**Table 1:** Inhibition of human protein kinase CK2 *in vitro* by indeno[1,2‐*b*]indoles and the reference compound CX‐4945. IC_50_ values were derived from dose–response curves with nine different inhibitor concentrations (*n* = 3).

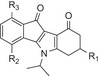					
Compound	R_1_	R_2_	R_3_	Inhibition (%) 10 µm	IC_50_ (µm)
5a‐1	‐CH_3_	‐H	‐OCH_3_	55	8.170
5a‐2	‐CH_3_	‐OCH_3_	‐H	100	0.025
5b‐1	‐CH_2_CH_3_	‐H	‐OCH_3_	50	9.910
5b‐2	‐CH_2_CH_3_	‐OCH_3_	‐H	100	0.047
CX‐4945				100	0.0037^a^

^a^ Previously reported by Gozzi et al.[38]

### CK2α expression patterns in different tumor cell lines

**Fig. 2:**
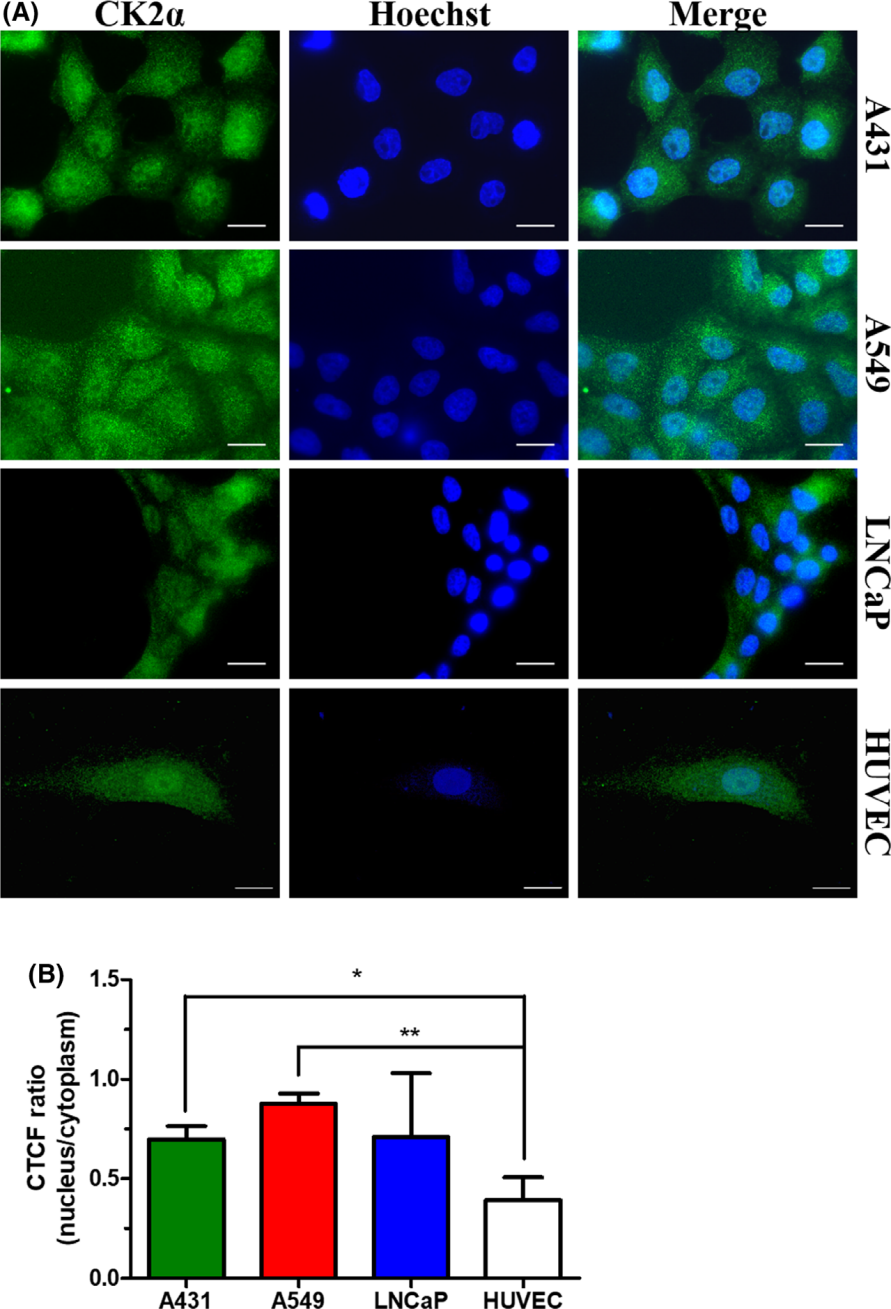
Expression pattern of CK2α in A431, A549, and LNCaP tumor cell lines and HUVECs. (A) Immunofluorescent CK2α signals (left, green) were detected using an anti‐CK2α antibody (1AD9, Santa Cruz Biotechnology, Santa Cruz, CA, USA). Hoechst 33342 was used to visualize the nuclei (middle, blue). Merged images are displayed on the right. Scale bar is 20 µm. (B) Quantitative analysis of fluorescence signals in the nucleus and cytoplasm of stained cells. Ratios of nuclear/cytoplasmic CTCF values from stained cells were determined. Data represent mean ± SD values from three randomly selected cells for each cell line. Statistical significance was analyzed using a two‐tailed unpaired Student’s *t*‐test, *P < 0.05, **P < 0.01.

The ability of both indeno[1,2‐*b*]indole derivatives **5a‐2** and **5b‐2** to strongly inhibit CK2 activity in our *in vitro* kinase assay, though less potent than CX‐4945, prompted us to examine their anti‐tumoral effects. For this purpose, we utilized three different tumor cell lines, namely A431 epidermoid cancer cells, A549 lung adenocarcinoma cells, and LNCaP prostate cancer cells. These cell lines were selected because they cover a broad spectrum of some of the most abundant types of cancers [49]. Tumor cells are known to overexpress CK2. Furthermore, the protein kinase is distributed throughout the whole cell, but particularly detectable in the nucleus [4]. Here, we examined the CK2α expression patterns in A431, A549, and LNCaP cells to verify, if these cell lines also exhibit the typical cancer cell characteristics and thus provided suitable cancer cell models for this study. In addition, non‐cancer cells (HUVEC) were included in this analysis to examine whether they demonstrate a different CK2 expression pattern. As shown in Fig. 2A, strong CK2α expression was found in all three cancer cell lines, with CK2α immunofluorescent signals being detectable in different cellular compartments. An increased fluorescence signal was particularly observed in the nuclei of A431, A549, and LNCaP cells (Fig. 2B). This indicates increased expression of CK2α in the nuclear compartments of these tumor cell lines. HUVECs showed a ubiquitous CK2α expression as well. However, in contrast to the tumor cells, no considerably high amount of CK2α could be detected in the nuclei of these non‐cancer cells (Fig. 2B).

Taken together, A431, A549, and LNCaP cells exhibited CK2α expression patterns typical of tumor cells, in contrast to HUVECs. Therefore, these three tumor cell lines should be suitable for studying cellular effects induced by CK2 inhibitors.

### Intracellular inhibition of CK2 activity

**Fig. 3:**
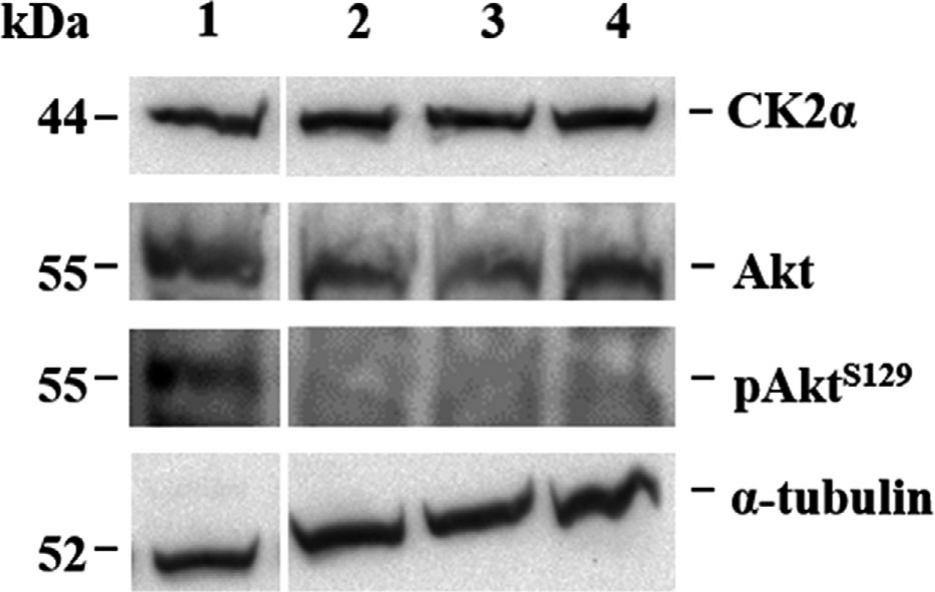
Inhibition of Akt phosphorylation by the indeno[1,2‐*b*]indoles 5a‐2 and 5b‐2. LNCaP cells were treated with either vehicle control (1) or 20 µm of 5a‐2 (2), 5b‐2 (3) or CX‐4945 (4) for 24 h. CK2α, Akt, pAkt^S129^, and α‐tubulin (loading control) were detected in lysates of treated cells via Western blot. LNCaP cell lysates used for blotting were prepared from an experiment with three replicates.

Following the determination of the activity of **5a‐2** and **5b‐2** as CK2 inhibitors *in vitro*, we investigated their ability to interfere with CK2 activity in cancer cells. Using the reference compound CX‐4945, for which the inhibition of intracellular CK2 activity has already been described [26], the effects of **5a‐2** and **5b‐2** should be evaluated. Here, LNCaP cells were treated with the indeno[1,2‐*b*]indoles as well as with the reference compound CX‐4945 at 20 µm for 24 h followed by western blot analysis of CK2‐specific phosphorylation of Akt at position serine 129 (pAkt^S129^) [50] in the lysates of the treated cells. Cells treated with 1% DMSO served as vehicle control. This analysis was performed as described by Schmitt et al. [45]. Our results showed a clear reduction in the intensity of pAkt^S129^ band in lysates from cells treated with CX‐4945, **5a‐2** or **5b‐2** compared to the vehicle control (Fig. 3). Notably, the applied treatments showed no effect on the level of CK2α expression in the lysates when compared to control cells, indicating no destabilizing effects on the protein in the cells as treated (Fig. 3). These results hint at the capability of both tested indeno[1,2‐*b*]indoles to interfere effectively with intracellular CK2 activity as demonstrated by their ability to block CK2‐specific phosphorylation of Akt in treated LNCaP cells analogous to CX‐4945.

**Fig. 4:**
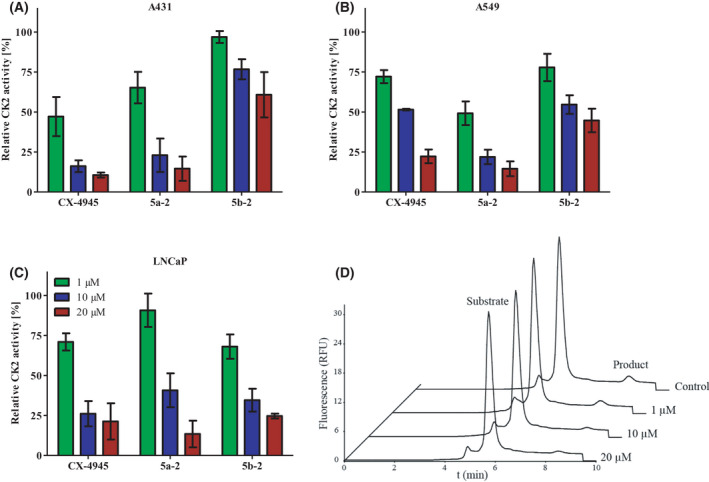
Inhibition of CK2 activity in cancer cells by compounds 5a‐2 and 5b‐2 as compared to CX‐4945. CK2 activity was determined in lysates from (A) A431, (B) A549, and (C) LNCaP cells using a CE‐based assay [39] and normalized to control (1% DMSO). Data represent mean ± SD, *n* = 3 with triplicate samples. (D) Representative electropherograms for the determination of CK2 activity in LNCaP cell lysates after treatment with CX‐4945 at the indicated concentrations.

To directly analyze the interaction of **5a‐2** and **5b‐2** with CK2 within the cellular matrix in a quantitative manner, a CE‐based analysis was utilized. Cultured A431, A549, and LNCaP cells were treated with either **5a‐2**, **5b‐2,** or CX‐4945 at 1, 10, and 20 µm for 24 h, while control wells received 1% DMSO. After cells were harvested and lysed, CK2 activity in the soluble fraction of each lysate was determined using a CE‐based kinase activity assay as described recently by El‐Awaad et al. [39]. As shown in Fig. 4, all tested compounds were able to reduce the intracellular CK2 activity concentration dependently. Both, **5a‐2** and the reference compound CX‐4945 reduced CK2 activity in all three cancer cell lines by more than 75% at 20 µm. Treatment with **5b‐2** led to a similar level of CK2 inhibition in LNCaP but less pronounced effects on A431 and A549 cells (40‐65% inhibition at 20 µm). These results confirm the ability of **5a‐2** to interfere directly and strongly with intracellular CK2 activity to a similar extent as CX‐4945, supporting our observations with regard to blocking of Akt phosphorylation in LNCaP cells. The ability of **5a‐2** to induce similar CK2 inhibition as CX‐4945 in all cell lines, despite the clear lower potency *in vitro* (IC_50_ CX‐4945 = 3.7 nm; IC_50_ **5a‐2** = 25 nm) suggests that the indeno[1,2‐*b*]indole inhibits CK2 more effectively within the cell than CX‐4945. The observed weaker CK2 activity inhibition by **5b‐2** compared to **5a‐2** is consistent with its lower potency toward CK2 inhibition as mentioned before in Table 1 (IC_50_ = 47 nm). In further studies, we therefore focused on **5a‐2** as the more potent CK2 inhibitor.

### Cellular uptake of CK2 inhibitors

The amount of the investigated CK2 inhibitors within the cell is critical for inhibiting the intracellular activity of the target. Thereby, an enhanced uptake could compensate for the differences in the inhibitory potency of **5a‐2** and CX‐4945 *in vitro*. To estimate the ability of the indeno[1,2‐*b*]indole and the reference compound to penetrate cells, their polarities were determined based on *n*‐octanol/water partition coefficients (LogD_7.4_) according to Galla et al. [43]. These values correlate with the passive diffusion of drugs through cell membranes [51]. Here, the CK2 inhibitors were dissolved in the aqueous phase and combined with the *n*‐octanol phase in three different ratios (1 : 1, 2 : 1, 1 : 2). Both phases were then thoroughly mixed before the concentration of the substances within the aqueous phase was quantified by HPLC‐MS/MS. From this, the LogD_7.4_ values for **5a‐2** and CX‐4945 were calculated using equation 1. With a LogD_7.4_ value of 3.78 ± 0.20 **5a‐2** exhibited lower polarity than CX‐4945 (LogD_7.4_ = 3.46 ± 0.21) (Table 2). Although LogD_7.4_ values below 5 generally suggest an appropriate membrane permeability according to Lipinski’s rule of five [52], these LogD_7.4_ values indicate a more effective cellular uptake by passive diffusion for **5a‐2** compared to CX‐4945.

**Table 2:** LogD_7.4_ values of 5a‐2 and CX‐4945. Data represent mean ± SD, *n* = 9.

Compound	LogD_7.4_
5a‐2	3.78 ± 0.20
CX‐4945	3.46 ± 0.21

**Fig. 5:**
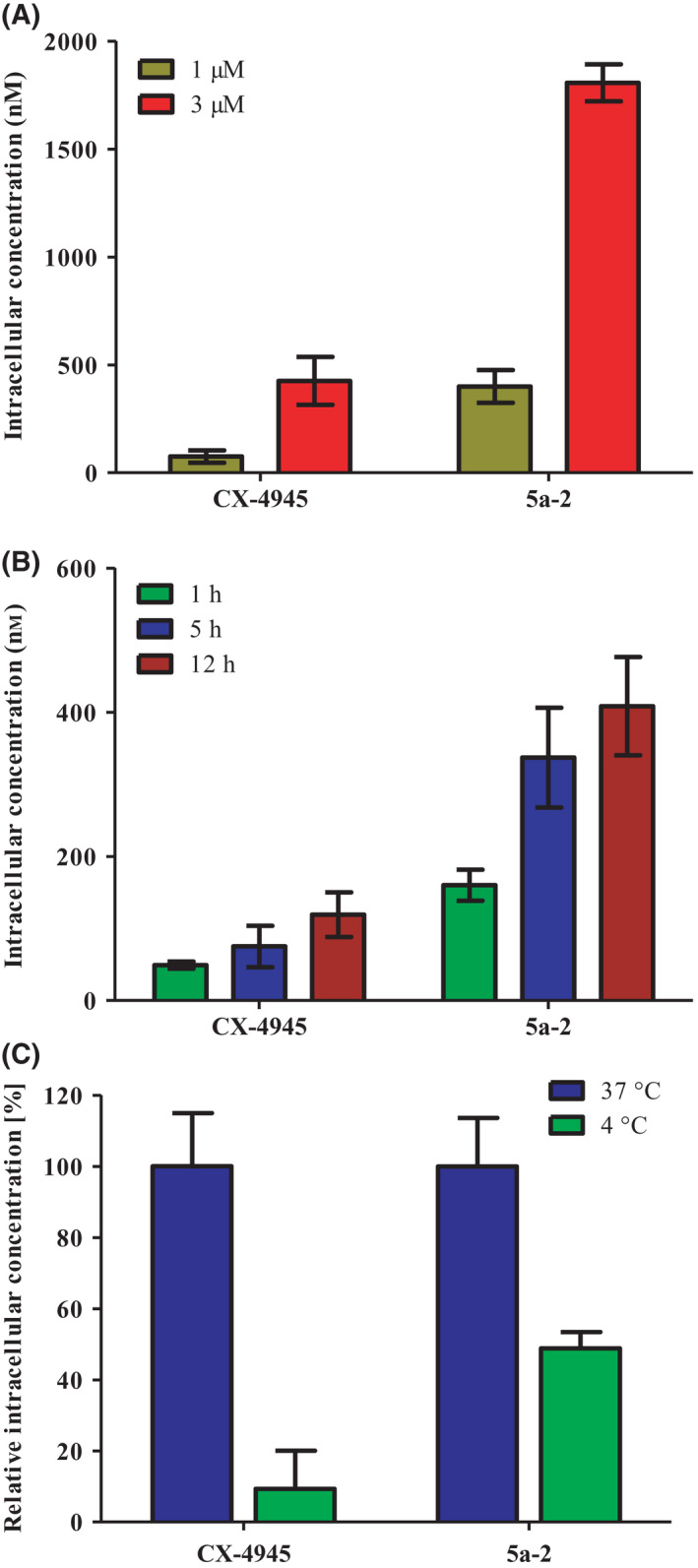
Cellular uptake of 5a‐2. (A) Extracellular concentration‐dependent uptake of the reference compound CX‐4945 and 5a‐2 into A431 cells. Cultured cells in 6‐well plates were incubated with either 1 or 3 µm of each compound followed by harvesting, cell counting, and lysis at 5 h post‐treatment. Quantitative analysis of the intracellular concentration (nm) for each compound was performed using HPLC‐MS/MS. (B) Time‐dependent uptake of the reference compound CX‐4945 and 5a‐2. Cells were cultured as described before and incubated with 1 µm of each compound for 1, 5, or 12 h. (C) Temperature‐dependent uptake of compounds CX‐4945 and 5a‐2. A431 cells were cultured in 6‐well plates as described before. After incubation with CK2 inhibitors at 1 µm for 3 h at 37 °C or 4 °C, intracellular concentrations were determined as described in (A). Data shown in (A), (B) and (C) represent mean ± SD, *n* = 3.

To analyze the cellular uptake of CK2 inhibitors in a quantitative manner, the intracellular concentrations of **5a‐2** and CX‐4945 were determined in A431 cells. Cultured cells were treated with inhibitors at 1 or 3 µm for 1, 5, or 12 h. Inhibitor concentrations in the lysates of treated cells were quantified using HPLC‐MS/MS. Intracellular concentrations were calculated as described elsewhere [48]. As shown in Fig. 5A‐B, CX‐4945 exhibited intracellular concentrations in the range of 49.2 ± 4.1 nm to 119.3 ± 25.4 nm after 1 h up to 12 h of treatment of A431 cells with the reference compound at 1 µm. In contrast, 160.8 ± 17.5 nm of **5a‐2** were detected in A431 cells treated with 1 µm of the compound already after 1 h. The concentration of the indeno[1,2‐*b*]indole increased to 408.3 ± 55.6 nm after 12 h. Consequently, intracellular concentrations of **5a‐2** were 2.4‐ to 3.4‐fold higher than those of CX‐4945. These results suggest a more efficient cellular uptake of **5a‐2** compared to that of CX‐4945. The high intracellular concentration of **5a‐2** could provide an explanation for the strong effect of **5a‐2** on intracellular CK2 activity, despite its higher IC_50_ value compared to CX‐4945.

To shed light on the mechanism of the cellular uptake for the studied CK2 inhibitors, their intracellular concentrations were determined after incubation at different temperatures. By lowering the incubation temperature to 4 °C, active transport processes are widely abolished. If a drug continues to be taken up cellularly at this temperature, this indicates passive diffusion as the dominant uptake mechanism. In case of a strong decrease of the intracellular drug concentration at 4 °C, its uptake by active transporters can be concluded [53]. To analyze the temperature‐dependent cellular uptake of **5a‐2** and CX‐4945, cultured A431 cells were treated with CK2 inhibitors at 1 µm for 3 h at 37 °C in a humidified atmosphere at 5% CO_2_ or at 4 °C under atmospheric conditions. Afterward, intracellular concentrations were quantified as described before. At 4 °C, the intracellular concentration of **5a‐2** was decreased by 51%. On the contrary, the lower incubation temperature led to a decrease in the intracellular concentration of CX‐4945 by 91% compared to that determined at 37 °C (Fig. 5C). These results indicate a higher influence of active transporters on the uptake of CX‐4945 than for the indeno[1,2‐*b*]indole and suggest that **5a‐2** is taken up mainly via passive diffusion. This is in agreement with the determined LogD_7.4_ values reflecting the lower polarity of the indeno[1,2‐*b*]indole. Since drugs are normally taken up faster by passive diffusion than by active transporters [53], this could explain the higher observed intracellular concentrations of **5a‐2** compared to the reference compound CX‐4945.

### Cellular effects of 5a‐2

#### Suppression of tumor cell proliferation

**Fig. 6:**
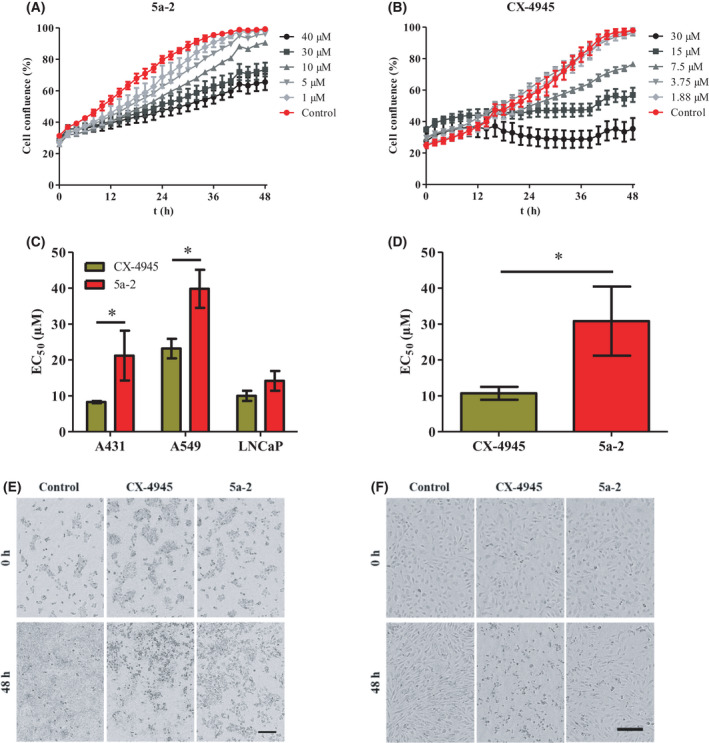
Anti‐proliferative effects of 5a‐2 compared to CX‐4945. Tumor cells were treated with either vehicle control (1% (v/v) DMSO) or CK2 inhibitors in at least eight different concentrations ranging from 0.47 to 50 µm. The % cell confluence was monitored for 48 h using IncuCyte® S3 live‐cell imaging system. (A‐B) Representative growth curves of A431 cells treated with vehicle control and 5a‐2 (A) or CX‐4945 (B) (mean ± SD, *n* = 3). (C) EC_50_ values of 5a‐2 and CX‐4945 represent the inhibitor concentration suppressing cell growth by 50% relative to control. (D) Suppression of HUVEC proliferation by 5a‐2 or CX‐4945. Data shown in (C) and (D) represent mean ± SD, *n* = 3 with triplicate samples. Statistical significance was analyzed using a two‐tailed unpaired Student’s *t*‐test, *P < 0.05. (E‐F) Representative phase‐contrast microscopy images of A431 cells (E) and HUVECs (F) treated with 30 µm of CX‐4945 or 5a‐2, immediately after treatment (0 h) and at 48 h post‐treatment using the IncuCyte® live‐cell imaging system, *n* = 3. Control cells were treated with vehicle (1% DMSO). Scale bar is 400 µm (E) or 200 µm (F).

Inhibition of intracellular CK2 activity by **5a‐2** suggested similar cellular effects of this indeno[1,2‐*b*]indole derivative and CX‐4945. Consideration of the cellular effects of CK2 inhibitors was primarily based on their impact on cancer and non‐cancer cell proliferation. Effects on tumor cell proliferation by **5a‐2** and CX‐4945 were examined in A431, A549, and LNCaP cells using an IncuCyte® S3 live‐cell imaging system (Sartorius). HUVECs were also analyzed as a representative model for non‐cancer cells. Here, **5a‐2** clearly suppressed the proliferation of the three tumor cell lines with EC_50_ values of 21.2 µm (A431), 39.8 µm (A549) and 14.2 µm (LNCaP) (Fig. 6A). Yet, CX‐4945 had significantly stronger effects on A431 and A549 proliferation compared to the indeno[1,2‐*b*]indole as evident by EC_50_ values of 8.3 µm and 23.2 µm. Thus, these EC_50_ values of the reference compound were 1.4‐ to 2.5‐fold lower than those of **5a‐2**. On the other hand, no significant differences between the EC_50_ values of CX‐4945 (10.0 µm) and **5a‐2** on LNCaP cells could be observed. Notably, the determined anti‐proliferative effects of CX‐4945 in our experiments were in the same range as reported in earlier studies. Siddiqui‐Jain et al. [25], for example, determined EC_50_ values between 1.71 and 20.01 µm for CX‐4945 on 43 different tumor cell lines. On the other hand, analysis of anti‐proliferative effects on HUVECs revealed EC_50_ values of 30.8 µm and 10.7 µm for **5a‐2** and CX‐4945, respectively (Fig. 6B). Thus, **5a‐2** showed 2.9‐fold lower anti‐proliferative effect against HUVECs compared to CX‐4945. This significantly lower effect of the indeno[1,2‐*b*]indole suggests a less nonspecific toxicity induced by **5a‐2**. These results, thus, demonstrate that **5a‐2** and CX‐4945, despite their similar effects on intracellular CK2 activity, differed in their anti‐proliferative effects.

#### Induction of apoptosis

**Fig. 7:**
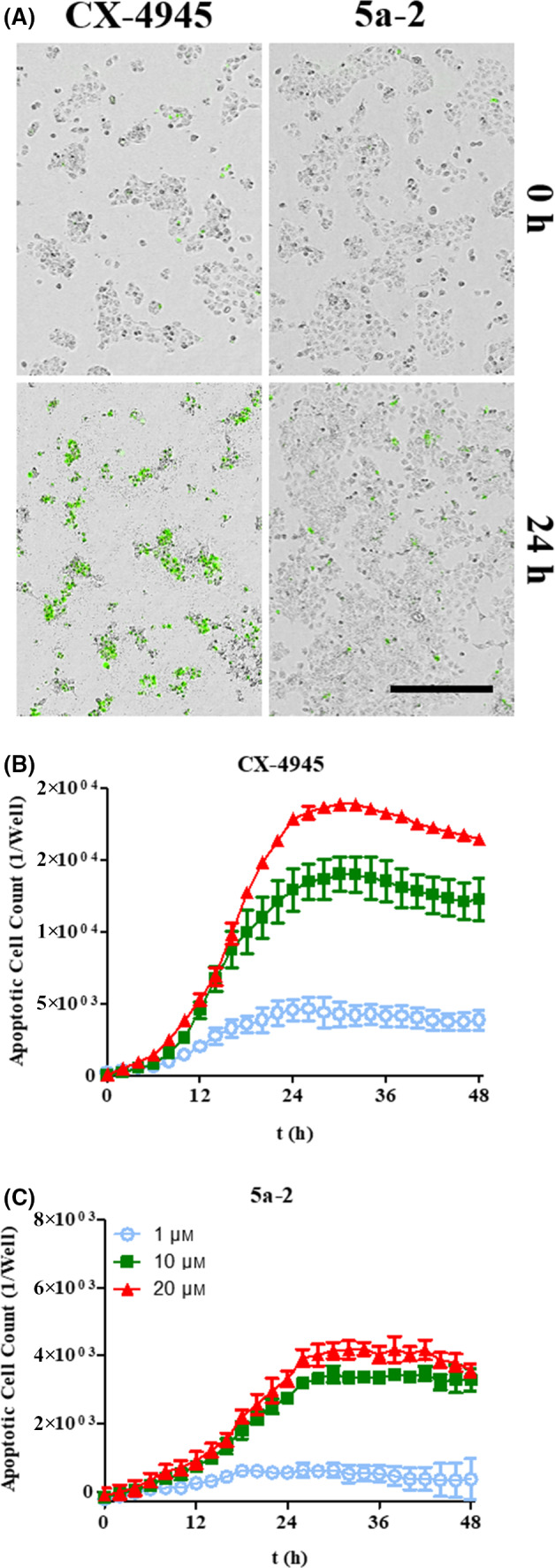
Pro‐apoptotic effects of 5a‐2 compared to CX‐4945. (A) Merge of phase‐contrast and fluorescence microscopy images of A431 cells immediately (0 h) and 48 h after treatment with 20 µm of the investigated CK2 inhibitors. Scale bar is 400 µm. (B‐C) Monitoring of apoptotic A431 cell count for 48 h following treatment with 1, 10, or 20 µm CX‐4945 (B) or 5a‐2 (C). Data represent mean ± SD, *n* = 3.

Since several studies reported that a decrease of CK2 activity in cancer cells leads to their apoptosis [20], we investigated the induction of this process in A431 cells after treatment with CK2 inhibitors. For this purpose, cells were monitored after treatment with **5a‐2** or CX‐4945 via IncuCyte® S3 live‐cell imaging system (Sartorius). Furthermore, a dye, which emits a green fluorescence upon cleavage by caspase 3/7 activity in eukaryotic cells during apoptosis (IncuCyte® Caspase‐3/7 dye, Sartorius), was applied to the cells. The fluorescence signals detected correlated with the number of apoptotic cells. As shown in Fig. 7, apoptosis was induced in A431 cells after treatment with CX‐4945 as well as with **5a‐2**. In this experiment, we determined up to 18,929 apoptotic cells per well after treatment with 20 µm CX‐4945. Treatment of A431 cells with **5a‐2** at 20 µm led to 4194 apoptotic cells per well. Therefore, the reference substance had a 4.5‐fold higher impact on the induction of apoptosis in A431 epidermal cancer cells than the indeno[1,2‐*b*]indole.

#### Reduction of tumor cell migration

**Fig. 8:**
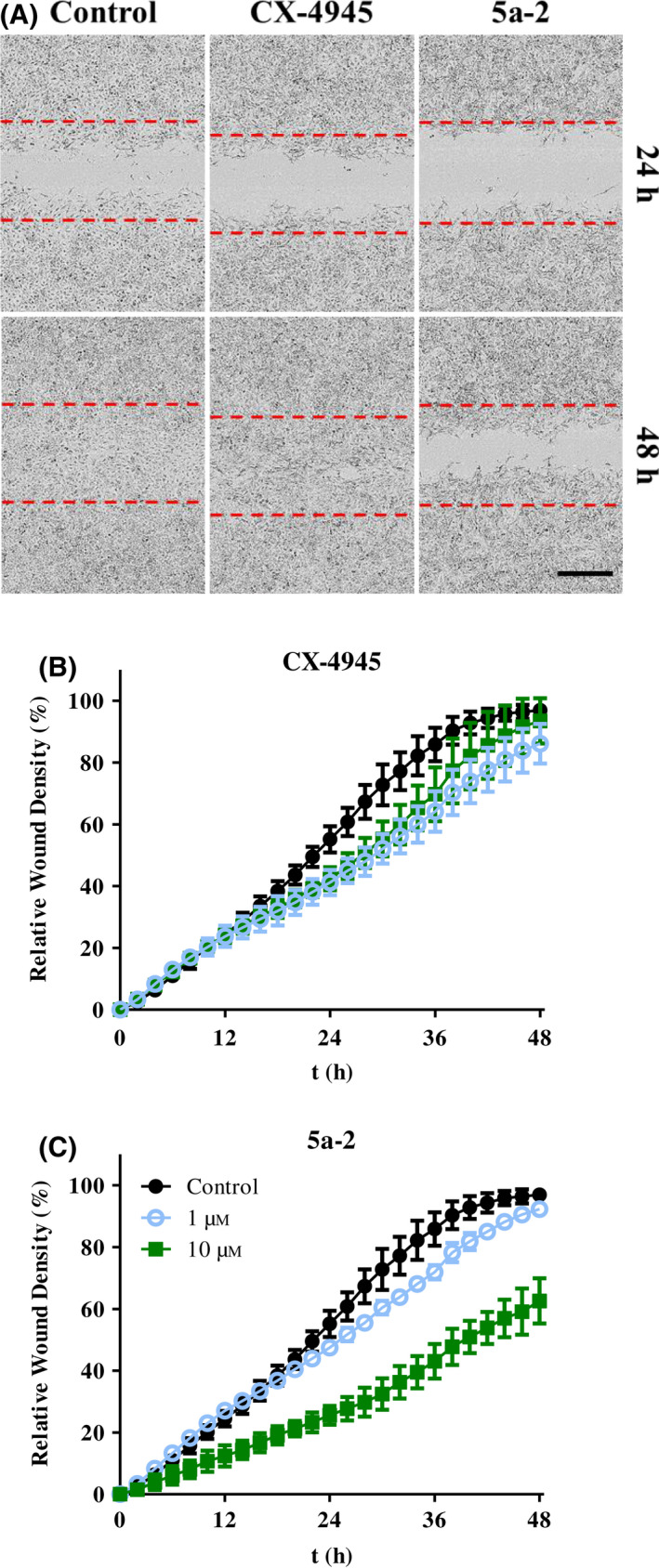
Anti‐migratory effects of 5a‐2 compared to CX‐4945. (A) Representative phase‐contrast microscopy images showing the reduction of A549 cell migration by 10 µm of the investigated CK2 inhibitors 24 h and 48 h post‐treatment. Control wells received 1% DMSO. Scale bar is 400 µm. (B‐C) Time‐dependent changes in wound density of A549 cells after treatment with vehicle control, 1 and 10 µm of CX‐4945 (B) or 5a‐2 (C). Data represent mean ± SD, *n* = 3.

Cellular migration is a process involving CK2 that plays a role in tumor metastasis [54, 55]. Therefore, reduction of migration of A549 lung cancer cells after treatment with **5a‐2** was investigated in this study. For this analysis, cells were cultured in 96‐well ImageLock plates (Sartorius) to a confluence of 100%. Afterward, a scratch wound was created in the cell layer of each well using an IncuCyte® WoundMaker tool (Sartorius). Then, cells were treated with **5a‐2** or CX‐4945 at 1 or 10 µm. The cell migration into the wound was determined using an IncuCyte® S3 Live Cell Imaging system (Sartorius). The results of this experiment (Fig. 8) showed a relative wound density of 93.5% 48 h after treatment with 10 µm CX‐4945. Cells treated with vehicle control reached a relative wound density of 96.9% after 48 h. After treatment with **5a‐2** at 10 µm, A549 cells reached a relative wound density of 62.6%. Thus, the indeno[1,2‐*b*]indole had a stronger effect on tumor cell migration than CX‐4945, which comes in contrast with our observation regarding the pro‐apoptotic effects of the investigated CK2 inhibitors. The anti‐migratory effects of the compounds were investigated on A431 cells as well (data not shown). Also in this case, **5a‐2** had a stronger effect than CX‐4945, further confirming the strong anti‐migratory effect of the indeno[1,2‐*b*]indole.

These observations raise the question whether variable degrees of inhibition of CK2 in cellular compartments by the studied inhibitors may be responsible for their preferential interference with cellular processes, that is, migration for **5a‐2** or apoptosis for CX‐4945.

### Subcellular distribution

**Fig. 9:**
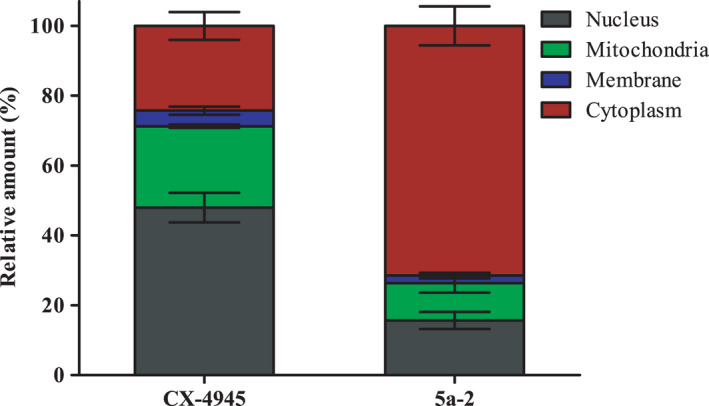
Subcellular distribution of CX‐4945 and 5a‐2. LNCaP prostate cancer cells were cultured in 75‐cm^2^ flasks to a confluence of 90%. After 5 h of treatment with 1 µm 5a‐2 or CX‐4945 cells were harvested and lysed. Subcellular fractions were separated using a differential centrifugation protocol. Inhibitors were detected via HPLC‐MS/MS in each fraction. AUCs of the analyte peaks were related to each other. The sum of the AUCs of all fractions was set as 100%. Data represent mean ± SD of triplicate samples.

As mentioned earlier, despite their equivalent influence on the intracellular CK2 activity (Fig. 4), **5a‐2** and CX‐4945 clearly differ in their pro‐apoptotic (Fig. 7) and anti‐migratory effects (Fig. 8). The reason for this could be attributed to the site of action of the CK2 inhibitors. For this reason, we studied the subcellular distribution of **5a‐2** and CX‐4945. Therefore, subcellular fractions of LNCaP cells were separated and collected after 5 h of treatment with 1 µm **5a‐2** or CX‐4945 via differential centrifugation. CK2 inhibitors were detected in each fraction using HPLC‐MS/MS. AUCs of the analyte peaks in each fraction were set in relation to each other to determine relative amount of the substances (Fig. 9). Here, we were able to detect an amount of 49% in the nuclear fraction for CX‐4945, whereas the levels in the mitochondrial and cytoplasmic fractions were almost equal at 23% and 24%, respectively. At 4%, the lowest proportion of CX‐4945 was detected in the membrane fraction. In contrast, **5a‐2** was mainly present in the cytoplasm showing a proportion of 71%. In the mitochondrial and membrane fraction, 11% and 2% of indeno[1,2‐*b*]indole were determined, respectively. The nuclear fraction of treated cells contained 16% of **5a‐2**. These results reveal a major divergence in the subcellular distribution of CX‐4945 and the indeno[1,2‐*b*]indole, suggesting that the subcellular distribution of CK2 inhibitors may be determinant for their cellular effects in cancer cells.

## Discussion

As noted earlier, human protein kinase CK2 remains a critical target in cancer research [56]. Almost ten years ago, the first small‐molecule CK2 inhibitor, namely CX‐4945, entered clinical trials. Even though the drug has shown promising results in tumor therapy [29], it remains the only small‐molecule CK2 inhibitor in clinical trials. With compound **5a‐2** we identified a potent, ATP‐competitive CK2 inhibitor that reduced the protein kinases activity in tumor cells by more than 75% at an applied concentration of 20 µm. **5b‐2**, an indeno[1,2‐*b*]indole derivative differing from **5a‐2** only in a substituent at position 7, showed weaker effects on inhibition of CK2 activity both *in vitro* and in the cellular matrix. Therefore, the methyl group of **5a‐2** seems to be more favorable than the ethyl group of **5b‐2** at the same position in terms of inhibitory activity of the compound. Interestingly, the inhibitory effect on intracellular CK2 activity by **5a‐2** was equivalent to that of CX‐4945, despite its higher IC_50_ value (IC_50_ **5a‐2** = 25 nm; IC_50_ CX‐4945 = 3.7 nm). This could be shown by the reduction of the CK2‐specific Akt phosphorylation in LNCaP treated with **5a‐2** and CX‐4945 as well as by the inhibition of intracellular CK2 activity in A431, A549, and LNCaP cells by both CK2 inhibitors. Since intracellular target inhibition is highly dependent on drug concentration within the eukaryotic cell [57], we considered different levels of cellular uptake to be compensating for IC_50_ value differences of the CK2 inhibitors. Here, we demonstrated that the uptake of **5a‐2** was more effective than that of CX‐4945, as indicated by 2.4‐ to 3.4‐fold higher intracellular concentrations of the indeno[1,2‐*b*]indole. Thus, these higher concentrations may explain why **5a‐2** and CX‐4945 affected intracellular CK2 activity to the same extent, despite their different inhibitory potential *in vitro*. Furthermore, we were able to show that the uptake of **5a‐2** was mainly driven by passive diffusion, while active transporters appeared to play a major role in the uptake of CX‐4945. This observation correlated with the lower polarity of the indeno[1,2‐*b*]indole, which was determined here from the LogD_7.4_ values of the compounds. Consistent with our observations, a correlation between LogP values and passive diffusion of drugs was previously reported [51]. Since drugs are taken up more rapidly by passive diffusion than by active transporters [53], this may account for the more effective cellular uptake of **5a‐2** compared with CX‐4945.

Consideration of the cellular effects of **5a‐2** and CX‐4945 revealed differences for both CK2 inhibitors, which did not reflect the equivalent intracellular inhibition of the protein kinase activity. Although both substances reduced the proliferation of cancer cells, this occurred to different extents. While we determined EC_50_ values of 8.3 µm (A431), 23.2 µm (A549), and 10.0 µm (LNCaP) for CX‐4945, these values were 21.2 µm (A431), 39.8 µm (A549), and 14.2 µm (LNCaP) in the case of **5a‐2**. The analysis of the effects of CK2 inhibitors on non‐cancer cells, performed in this study using HUVECs as an example, revealed a smaller effect of **5a‐2** than of CX‐4945. The indeno[1,2‐*b*]indole suppressed HUVEC proliferation with an EC_50_ value of 30.8 µm, while the reference compound exhibited an EC_50_ value of 10.7 µm. This anti‐proliferative effect of CX‐4945 on non‐cancer cells has been observed in previous studies [58, 59]. The significantly higher EC_50_ value of **5a‐2** compared to CX‐4945 may indicate a less nonspecific cytotoxicity of the indeno[1,2‐*b*]indole. Recently, Wells et al. described a potent, highly selective CK2 inhibitor, which did not suppress tumor cell proliferation to the same extent of CX‐4945. This observation led Wells et al. to hypothesize that the potent anti‐proliferative effect of CX‐4945 may not be due to inhibition of CK2 alone but rather to off‐target effects. This hypothesis was supported by the inhibitory effect of CX‐4945 on the activity of other kinases [60]. Off‐target effects of CX‐4945 may provide a possible reason for its strong anti‐proliferative effect and thereby may explain the differences in the EC_50_ values of the reference compound and **5a‐2** in this study. To verify this assumption, the selectivity of the inhibitory effect of indeno[1,2‐*b*]indole must also be tested in future studies.

The strong pro‐apoptotic effect of CX‐4945 noted here is also consistent with observations of previous studies [48, 60]. Treatment of A431 cells with **5a‐2** also induced apoptosis, but to a much weaker extent. On the other hand, **5a‐2** reduced the migration of A549 and A431 cells more potently than CX‐4945. As part of tumor metastasis, migration is a cellular process that has previously been described as sensitive to CX‐4945 treatment [34, 55, 61]. The even stronger effect of **5a‐2** in this regard may represent an advantage of indeno[1,2‐*b*]indole over the reference compound in the treatment of systemic cancers [62]. Nevertheless, the question arises as to why the cellular effects of **5a‐2** and CX‐4945 differ despite equivalent inhibition of intracellular CK2 activity. One theory for these distinct cellular effects of the two CK2 inhibitors could be their site of action, since it is known that CK2 is involved in several processes taking place in different cellular compartment [11]. Analysis of subcellular CK2 inhibitor distribution showed that CX‐4945 was mainly present in the nuclear fraction, accounting for 50%. In contrast, the largest proportion of **5a‐2** was detected in the cytoplasm at 70%. Previous studies [4, 13] as well as the results obtained here showed a ubiquitous expression as well as an increased presence of CK2 in the nuclei of tumor cells. The protein kinase is known to be involved in apoptotic processes which take place in the nuclear matrix [63]. Furthermore, it could be shown that the inhibition of CK2 activity in the nuclei of cancer cells leads to their apoptosis [17]. Thus, the obtained high amount of CX‐4945 in the nuclear fraction may be responsible for its high impact on apoptosis induction. Recently, an interaction of CK2 and the chromatin remodeling enzyme Brg1 within the nucleus of proliferating myoblasts was observed [64]. Since this interaction takes place in the nucleus, the high amount of CX‐4945 in this fraction could be explanatory for its strong anti‐proliferative effects. On the other hand, it was observed that CK2 is involved in tumor cell migration by interacting with cytoplasmic proteins [7, 65]. Ca^2+^‐activated ion channels represent a class of transmembrane proteins that are involved in tumor cell migration [66]. Recently, the activity of the Ca^2+^‐activated ion channel TMEM16A could be shown to be dependent on phosphorylation by CK2 [67]. Therefore, the high amount of **5a‐2** within the cytoplasmic fraction could be the reason for CK2 inhibition at site, resulting in a loss of activation of proteins involved in tumor cell migration like TMEM16A.

In conclusion, we identified a 4,5,7‐trisubstituted indeno[1,2 *b*]indole as potent new CK2 inhibitor based on the results obtained in this study. Most notably, **5a‐2** was shown to inhibit the intracellular CK2 activity equivalent to CX‐4945, despite a lower inhibitory potential *in vitro*. Interestingly, **5a‐2** and CX‐4945 differed in the extent of their cellular effects and subcellular distribution. This led us to the theory that these effects are dependent on the site of action of CK2 inhibition. With this study, we present an approach to elucidate biological and pharmacological processes based on comprehensive investigation of effects of CK2 inhibitors on cancer cells. On this basis, insights could be gained to better understand the different roles of CK2 in cancer cell biology and ultimately help to develop new anti‐cancer drugs.

## Conflict of interest

The authors declare no conflict of interest. The authors alone are responsible for the content and writing of this article.

## Author contribution

RB and EE planned experiments; RB, LB, FA, LE, and CG performed experiments; RB, EE, and DA analyzed data; MLB supervised the chemical synthesis part of the project; RB and EE wrote the paper; JJ supervised and coordinated the project, critically analyzed the data, and designed the concept of the manuscript. All authors read and approved the final manuscript submitted for publication.

## Supporting information


**Scheme S1**. Chemical synthesis reagents and conditions.
**Fig. S1**. ^1^H NMR of compound **5a‐1** – CDCl_3_.
**Fig. S2**. ^13^C NMR of compound **5a‐1** – CDCl_3_.
**Fig. S3**. ^1^H NMR of compound **5a‐2** – CDCl_3_.
**Fig. S4**. ^13^C NMR of compound **5a‐2** – CDCl_3_.
**Fig. S5**. ^1^H NMR of compound **5b‐1** – CDCl_3_.
**Fig. S6**. ^13^C NMR of compound **5b‐1** – CDCl_3_.
**Fig. S7**. ^1^H NMR of compound **5b‐2** – CDCl_3_.
**Fig. S8**. ^13^C NMR of compound **5b‐2** – CDCl_3_.

## Data Availability

The data that support the findings of this study are available from the corresponding author upon reasonable request.
