# Supplementary material for: 4,5,7‐Trisubstituted indeno[1,2‐b]indole inhibits CK2 activity in tumor cells equivalent to CX‐4945 and shows strong anti‐migratory effects
Source: FEBS Open Bio. 2021 Dec 18;12(2):394–411. doi: 10.1002/2211-5463.13346 (PMC8804612; doi:10.1002/2211-5463.13346)
Supplement: Supplementary file 1 — Scheme S1. Chemical synthesis reagents and conditions. Fig. S1. 1H NMR of compound 5a‐1 – CDCl3. Fig. S2. 13C NMR of compound 5a‐1 – CDCl3. Fig. S3. 1H NMR of compound 5a‐2 – CDCl3. Fig. S4. 13C NMR of compound 5a‐2 – CDCl3. Fig. S5. 1H NMR of compound 5b‐1 – CDCl3. Fig. S6. 13C NMR of compound 5b‐1 – CDCl3. Fig. S7. 1H NMR of compound 5b‐2 – CDCl3. Fig. S8. 13C NMR of compound 5b‐2 – CDCl3. [file FEB4-12-394-s001.docx]

**SUPPORTING INFORMATION**

**4,5,7-Trisubstituted Indeno[1,2-*b*]indole Inhibits CK2 Activity in Tumor Cells Equivalent to CX-4945 and Shows Strong Anti-Migratory Effects**

Robin Birus^1^, Ehab El-Awaad^1,2^, Laurens Ballentin^1^, Faten Alchab^3^, Dagmar Aichele^1^
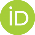
, Laurent Ettouati^4^
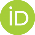
, Claudia Götz^5^, Marc Le Borgne^6^
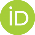
 and Joachim Jose^1,*^
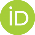


1 Institute of Pharmaceutical and Medicinal Chemistry, PharmaCampus, Westfälische Wilhelms-Universtität Münster, 48149 Münster, Germany

2 Department of Pharmacology, Faculty of Medicine, Assiut University, 71515, Egypt

3 EA 4446 Bioactive Molecules and Medicinal Chemistry, Faculté de Pharmacie-ISPB, SFR Santé Lyon-Est CNRS UMS3453-INSERM US7, Université Claude Bernard Lyon 1, Université de Lyon, F-69373 Lyon, France

4 CNRS UMR 5246 Institut de Chimie et Biochimie Moléculaires et Supramoléculaires (ICBMS), Faculté de Pharmacie, ISPB, Université Lyon 1, Université de Lyon, 8 Avenue Rockefeller, F-69373, Lyon, Cedex 08, France

5 Medical Biochemistry and Molecular Biology, Saarland University, D-66424 Homburg, Saarland, Germany

6 Small Molecules for Biological Targets Team, Centre de recherche en cancérologie de Lyon, Centre Léon Bérard, CNRS 5286, INSERM 1052, Université Claude Bernard Lyon 1, Univ Lyon, Lyon, 69373, France

**Table of Contents**

Chemical pathway for the synthesis of target compounds………………………..…….....…2

Detailed experimental procedure for synthesis of target compounds…………….…….……3

Spectral data of target compounds……………...…..………………………….……………10

References…………………………………………………………………...………………18

***Chemical pathway for the synthesis of target compounds***

The four tetrahydroindeno[1,2-*b*]indole-9,10-diones **5a,b** were synthesized according to previously reported methods^1-3^ as shown in Scheme S1. 5-Ethyl-3-isopropylamino-cyclohex-2-enone (**2b**) was initially obtained by the method used for **2a** ^4^. The 1 or 4-methoxyindenoindoles **5a,b** were obtained in two steps. Condensation of the enaminones **2a,b** with 4-methoxyninhydrin **3** led to the dihydroxylated derivatives **4a,b** (not separated at this point) which were then deoxygenated using the tetraisopropylthionylamide (TIPTA) to afford target compounds **5a,b**.

**Scheme S1.** Chemical synthesis reagents and conditions: (a) R_2_NH_2_, toluene, reflux, 4 to 6 h; (b) MeOH, rt, 22 h; (c) (*i*Pr_2_N)_2_SO (TIPTA), DMF, AcOH, rt, 22 h.

***Detailed experimental procedure for synthesis of target compounds***

**5-Ethyl-3-isopropylamino-cyclohex-2-enone (2b)**

Isopropylamine (0.39 g, 6.70 mmol) and 5-ethyl-cyclohexane-1,3-dione (1 g, 6.70 mmol) were heated in 15 mL toluene at reflux for 4 h. The title product was recovered as a yellow powder.

**Yield:** 82%. **MP:** 78 °C.

**IR (KBr υ_max_ cm^-1^):** 3249 (N-H), 1533 (C=O).

**^1^H NMR (DMSO-d6_,_ δ ppm, *J* Hz):**

The following abbreviations are used: s: singlet; bs: broad singlet; d: doublet; t: triplet; dd: doubled doublet; m: multiplet. NMR analysis of compounds was performed with the same experiments as previously described ^5^.

6.85 (d, 1H, *J =* 6.8, N-H), 4.78 (s, 1H, H-2), 3.46 (m, 1H, CHMe_2_), 2.33 (d, 1H, *J* = 16, H-4 or H-6), 2.11 (d, 1H, *J* = 13.2, H-4 or H-6), 2.02 (m, 1H, H-4 or H-6), 1.83 (d, 1H, *J* = 11.6, H-4 or H-6), 1.78 (m, 1H, H-5) 1.30 (2H, m, CH_2-_Et), 1.09 (d, *J* = 5.2, 3H, NCHMe_2_), 1.08 (d, *J* = 5.6, 3H, NCHMe_2_), 0.86 (t, 3H, *J* = 7.2, CH_3_-Et).

**^13^C NMR (DMSO-d6, δ ppm):** 194.0 (C-1), 162.2 (C-3), 94.3 (C-2), 43.2 (NCHMe_2_), 42.7 (C-6), 35.3 (C-5), 34.5 (C-4), 27.8 (CH_2-_Et), 21.6 (CH_3_), 21.5 (CH_3_), 11.0 (CH_3_-Et).

**MS-ESI^+^ (m/z):** 182.2 [M + H]^+^.

**Mixture of 4b,9b-dihydroxy-5-isopropyl-1-methoxy-7-methyl-4b,5,6,7,8,9b-hexahydro-indeno[1,2-*b*]indole-9,10-dione (4a-1) and 4b,9b-dihydroxy-5-isopropyl-4-methoxy-7-methyl-4b,5,6,7,8,9b-hexahydro-indeno[1,2-*b*]indole-9,10-dione (4a-2)**

Enaminone **2a** (0.1 g, 0.6 mmol) and 4-methoxyninhydrin **3** (0.46 g, 0.6 m mol) were dissolved in 3 mL of methanol and the reaction mixture was stirred at room temperature for 22 h during which the reaction time was monitored by TLC (CH_2_Cl_2_/acetone 1:2). After work up, title products were recovered as two regioisomers **4a-1** and **4a-2** (yellow powder). At this stage, it was not possible to separate them.

**Yield:** 92%.

**IR (KBr υ_max_ cm^-1^):** 3385 (OH), 3266 (OH), 1716 (C=O), 1590 (C=O), 1272 (C-OMe).

**MS-ESI^+^ (*m/z*):** 380.2 [M + Na]^+^, 358.2 [M + H]^+^.

**Mixture of 7-ethyl-4b,9b-dihydroxy-5-isopropyl-1-methoxy-4b,5,6,7,8,9b-hexahydro-indeno[1,2-*b*]indole-9,10-dione (4b-1) and 7-ethyl-4b,9b-dihydroxy-5-isopropyl-4-methoxy-4b,5,6,7,8,9b-hexahydro-indeno[1,2-*b*]indole-9,10-dione (4b-2)**

Enaminone **2b** (0.3 g, 1.65 mmol) and 4-methoxyninhydrin **3** (0.344 g, 1.65 mmol) was introduced in 5 mL of methanol and the reaction mixture was stirred at room temperature for 22 h, during which the reaction time was monitored by TLC (CH_2_Cl_2_/acetone 1:2). After work up, title products were recovered as two regioisomers **4b-1** and **4b-2** (yellow powder). At this stage, it was not possible to separate them.

**Yield:** 97%.

**IR (KBr υ_max_ cm^-1^):** 2965 (CH), 1718 (C=O), 1591 (C=O), 1274 (C-OMe).

**MS-ESI^+^ (*m/z*):** 372.2 [M + H]^+^.

**5-Isopropyl-1-methoxy-7-methyl-5,6,7,8-tetrahydro-indeno[1,2-*b*]indole-9,10-dione (5a-1) and 5-isopropyl-4-methoxy-7-methyl-5,6,7,8-tetrahydro-indeno[1,2-*b*]indole-9,10-dione (5a-2)**

Mixture of **4a-1** and **4a-2** (0.2 g, 0.56 mmol) was dissolved in 0.88 mL DMF and 0.17 mL acetic acid was added. TIPTA (0.35 g, 1.40 mmol) then was added. A precipitate appeared after 22 h. After work-up, the product was obtained as a mixture of two regioisomers **5a-1** and **5a-2**. They were separated by flash chromatography (CH_2_Cl_2_ /acetone 1:2).

***5-Isopropyl-1-methoxy-7-methyl-5,6,7,8-tetrahydro-indeno[1,2-b]indole-9,10-dione (5a-1)***

Orange powder.

**Yield:** 69%. **Mp:** 244 °C.

**H^1^ NMR (CDCl_3_, δ ppm, *J* H_Z_):** 7.20 (dd, 1H, *J* = 7.3 and *J* = 8.7, Ar-H), 6.76 (m, 2H, Ar-H), 4.60 (sept, *J* = 7.1 1H, CHMe_2_), 3.90 (s, 3H, OMe), 2.90 (dd, 1H, *J*= 4.3, and *J* = 15.9, H-8), 2.53-2.43 (m, 2H, H-6 and H-8), 2.36 (m, 1H, H-7), 2.17 (m, 1H, H-6), 1.63 (d, *J* = 6.1, 3H, NCH(Me)_2_), 1.61 (d, *J* = 6.2, 3H, NCH(Me)_2_), 1.15 (d, 3H, *J* = 6.5, CH_3_).

**^13^C NMR (CDCl_3_, δ ppm):** 191.7 (C-9), 183.2 (C-10), 157.5 (C-1), 149.5 (C-4b), 148.2 (C-5a), 137.7 (C-4a), 134.22 (C-3), 125.1 (C-10a), 122.7 (C-9b), 121.1 (C-4b), 117.30 (C-9a), 114.6 (C-2), 112.4 (C-4), 56.2 (OCH_3_), 49.3 (N-CH(Me)_2_), 46.2 (C-8), 31.9 (C-6), 31.1 (C-7), 22.9 (CH_3_), 21.7 (CH_3_), 21.3 (CH_3_).

**MS-ESI^+^ (*m/z*):** 668.7 [2 M + Na]^+^, 345.9 [M + Na]^+^, 323.9 [M + H]^+^.

**HRMS-ESI^+^ (*m/z*):** [M+Na^+^] calcd for C_20_H_21_NNaO_3_ 346.1414; found 346.1407.

***5-Isopropyl-4-methoxy-7-methyl-5,6,7,8-tetrahydro-indeno[1,2-b]indole-9,10-dione (5a-2)***

Orange powder

**Yield:** 31%. **Mp:** 239 °C.

**H^1^ NMR (CDCl_3_, δ ppm, *J* H_Z_):** 7.09-7.04 (m, 2H, 2 Ar-H), 6.86 (dd, 1H, *J* = 1.8 and 7.6, Ar-H), 6.71 (d, 1H, *J* = 8.6, Ar-H), 5.67 (br s, 1H, CHMe_2_), 3.87 (s, 3H, OMe), 2.99 (dd, 1H, *J*= 4.1 and 16.1, H-8), 2.61-2.47 (m, 2H, H-6 and H-8), 2.33 (m, 1H, H-7), 2.16 (m, 1H, H-6), 1.55 (d, 3H, *J* = 6.9, NCH(Me)_2_, 1.53 (d, 3H, *J* = 6.9, NCH(Me)_2_, 1.13 (d, 3H, *J* =6.6, CH_3_).

**^13^C NMR (CDCl_3_, δ ppm):** 191.9 (C-9), 183.9 (C-10), 153.8 (C-4), 149.6 (C-4b), 148.5 (C-5a), 140.4 (C-10a or C-4a), 130.0 (C-2), 122.0 (C-9b), 118.5 (C-9a), 117.6 (C-1 or C-3), 116.9 (C-3 or C-1), 56.0 (OCH_3_), 50.9 (N-CH(Me)_2_), 46.1 (C-8), 33.5 (C-6), 31.4 (C-7), 22.3 (CH_3_), 21.7 (CH_3_), 21.1 (CH_3_).

**MS-ESI^+^ (*m/z*):** 669.1 [2 M + Na]^+^, 346.1 [M + Na]^+^, 324.1 [M + H]^+^.

**HRMS-ESI^+^ (*m/z*):** [M+Na^+^] calcd for C_20_H_21_NNaO_3_ 346.1414; found 346.1407.

**7-Ehyl-5-isopropyl-1-methoxy-5,6,7,8-tetrahydro-indeno[1,2-*b*]indole-9,10-dione (5b-1) and 7-ethyl-5-isopropyl-4-methoxy-5,6,7,8-tetrahydro-indeno[1,2-*b*]indole-9,10-dione (5b-2)**

A mixture of **4b-1** and **4b-2** (0.5 g, 1.34 mmol) was dissolved in 2.20 mL DMF and 0.43 mL acetic acid. TIPTA (0.83 g, 3.37 mmol) was then added. A precipitate appeared after 22 h. After work-up, the product was obtained as a mixture of two regioisomers **5b-1** and **5b-2**. They were separated by flash chromatography (CH_2_Cl_2_ /acetone: 1:2).

***7-Ethyl-5-isopropyl-1-methoxy-5,6,7,8-tetrahydro-indeno[1,2-b]indole-9,10-dione (5b-1)***

Orange powder.

**Yield:** 67%. **Mp:** 226 °C.

**IR (KBr υ_max_ cm^-1^):** 1702 (C=O), 1660 (C=O), 1259 (C-OMe).

**^1^H NMR (CDCl_3,_ δ ppm, *J* Hz):** 7.20 (dd, *J =* 7.3 and 8.5, 1H, Ar-H), 6.78-6.73 (m, 2H, Ar-H), 4.61 (sept, *J* = 6.9, 1H, CHMe_2_), 3.90 (s, 3H, OMe), 2.91 (dd, 1H, *J* = 3.7 and *J* = 16.1, H-8), 2.57 (m, 1H, H-6), 2.47 (m, 1H, H-7), 2.20-2.13 (m, 2H, H-6 and H-8), 1.62 (t, 6H, *J* = 7.2, NCH(Me)_2_), 1.50 (m, 2H, CH_2-_Et), 0.98 (d, 3H, *J* = 7.4, CH_3_).

**^13^C NMR (CDCl_3_, δ ppm):** 191.9 (C-9), 183.4 (C-10), 157.5 (C-1), 149.5 (C-4b), 148.3 (C-5a), 137.7 (C-4a), 134.2 (C-3), 129.7 (C-10a), 123.1 (C-9b), 122.6 (C-4b), 117.4 (C-9a), 114.5 (C-2), 112.4 (C-4), 56.2 (OCH_3_), 49.2 (N-CH(Me)_2_), 44.0 (C-8), 37.6 (C-7), 29.6 (CH_2_-Et), 28.5 (C-6), 21.9 (CH_3_), 21.7 (CH_3_), 11.3 (CH_3_).

**MS-ESI^+^ (*m/z*):** 697.4 [2 M + Na]^+^, 360.2 [M + Na]^+^, 338.2 [M + H]^+^.

**HRMS-ESI^+^ (*m/z*):** [M+Na^+^] calcd for C_21_H_23_NNaO_3_ 360.1570; found 360.1558.

***7-Ethyl-5-isopropyl-4-methoxy-5,6,7,8-tetrahydro-indeno[1,2-b]indole-9,10-dione (5b-2)***

Orange powder

**Yield:** 33%. **Mp:** 203 °C.

**IR (KBr υ_max_ cm^-1^):** 1702 (C=O), 1664 (C=O), 1267 (C-OMe).

**^1^H NMR (CDCl_3,_ δ ppm, *J* Hz**)**:** 7.14-7.08 (m, 2H, 2 Ar-H), 6.88 (dd, 1H, *J* = 1.6 and *J* = 7.5, Ar-H), 5.70 (br s, 1H, CHMe_2_), 3.89 (s, 3H, OMe), 3.19 (m, 1H, H-7), 2.79-2.69 (m, 2H, H-6 and H-8), 2.38-2.24 (m, 2H, H-8 and H-6), 1.73 (t, 6H, *J*= 7.3, NCH(Me)_2_), 1.66 (m, 2H, CH_2-_Et), 1.13 (t, 3H, *J* = 7.3, CH_3_).

**^13^C NMR (CDCl_3_, δ ppm):** 192.0 (C-9), 183.9 (C-10), 153.8 (C-4), 149.6 (C-4b), 148.6 (C-5a), 140.4 (C-10a or C-4a), 130.1 (C-2), 122.0 (C-9b), 118.7 (C-9a), 117.6 (C-1 or C-3), 117.0 (C-3 or C-1), 55.9 (OCH_3_), 50.9 (N-CH(Me)_2_), 43.9 (C-8), 38.0 (C-7), 31.2 (CH_2_-Et), 28.3 (C-6), 22.3 (CH_3_), 21.7 (CH_3_), 11.2 (CH_3_-Et).

**MS-ESI^+^ (*m/z*):** 697.4 [2 M + Na]^+^, 338.2 [M + H]^+^.

**HRMS-ESI^+^ (*m/z*):** [M+Na^+^] calcd for C_21_H_23_NNaO_3_ 360.1570; found 360.1555.

***Spectral data of target molecules***

**Figure S1.** ^1^H NMR of compound **5a-1** – CDCl_3_


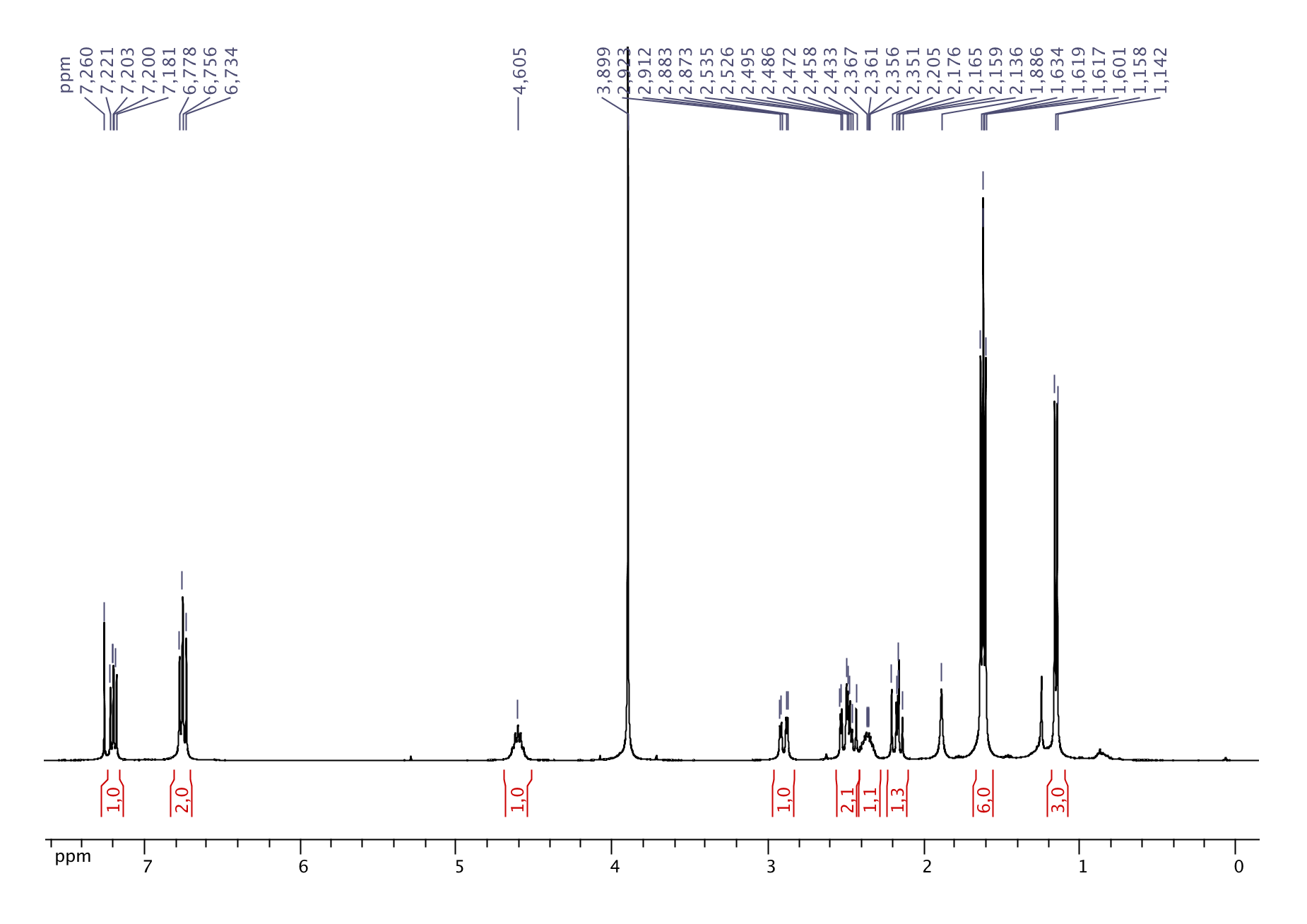


**Figure S2.** ^13^C NMR of compound **5a-1** – CDCl_3_


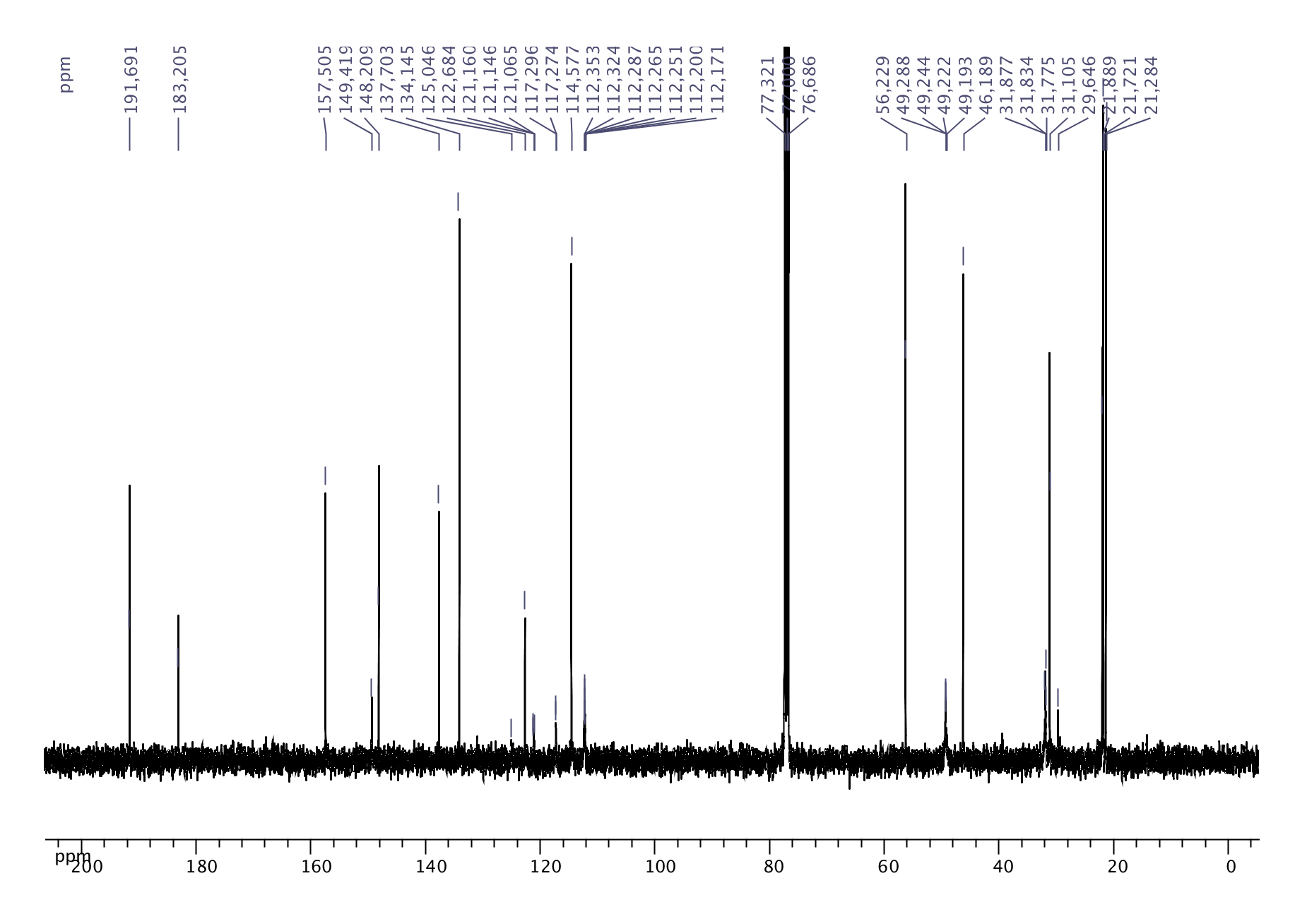


**Figure S3.** ^1^H NMR of compound **5a-2** – CDCl_3_


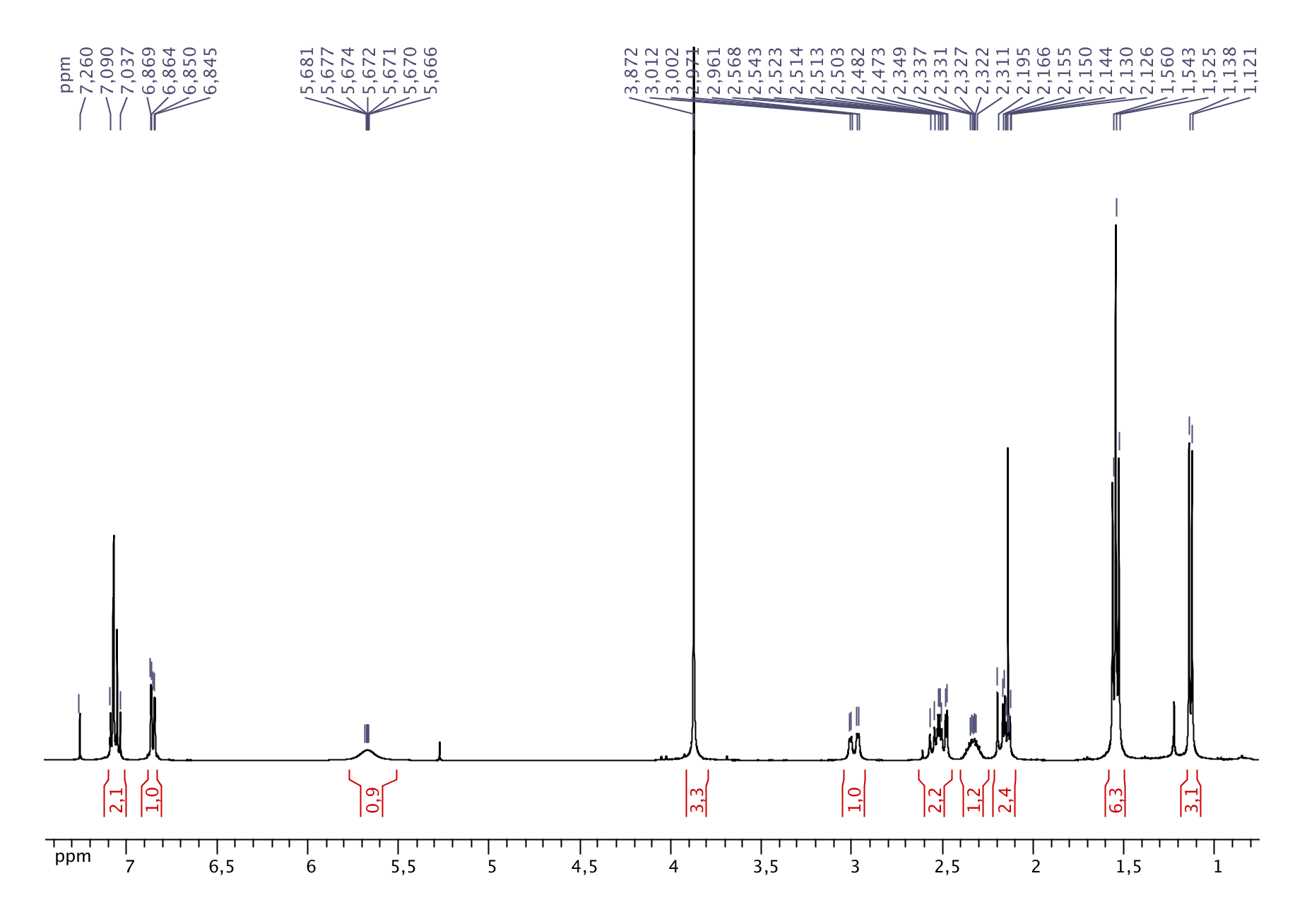


**Figure S4.** ^13^C NMR of compound **5a-2** – CDCl_3_


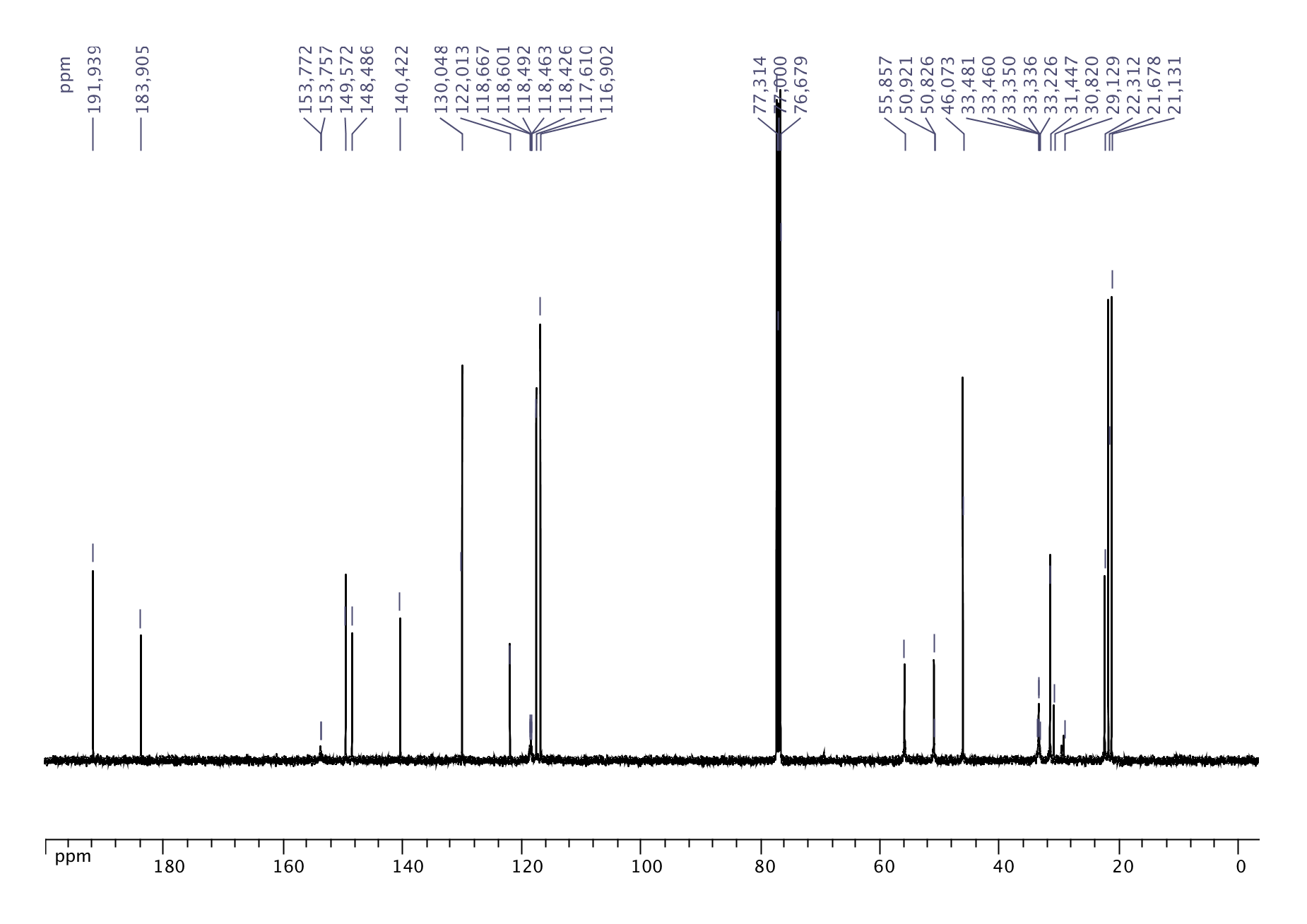


**Figure S5.** ^1^H NMR of compound **5b-1** – CDCl_3_


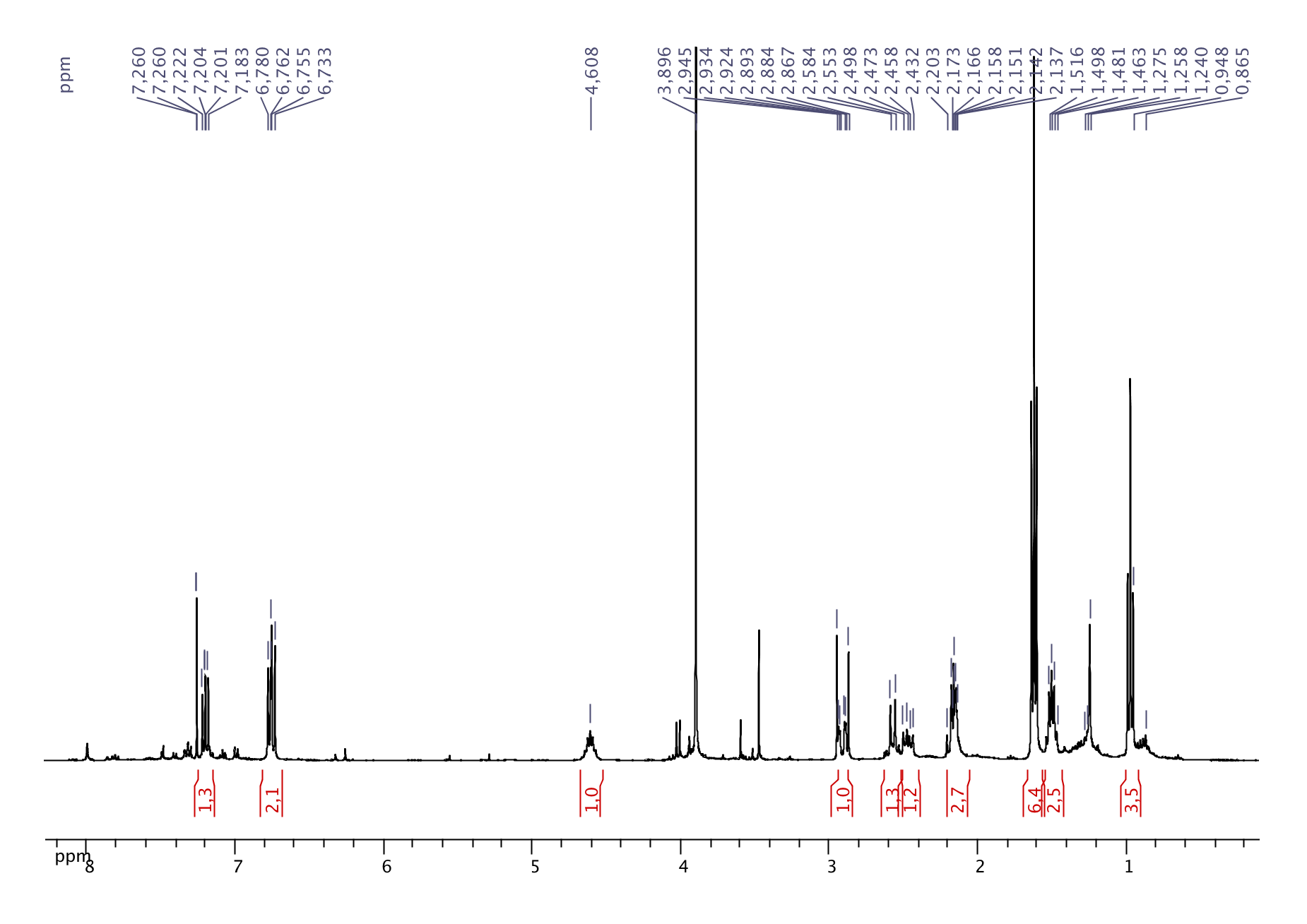


**Figure S6.** ^13^C NMR of compound **5b-1** – CDCl_3_


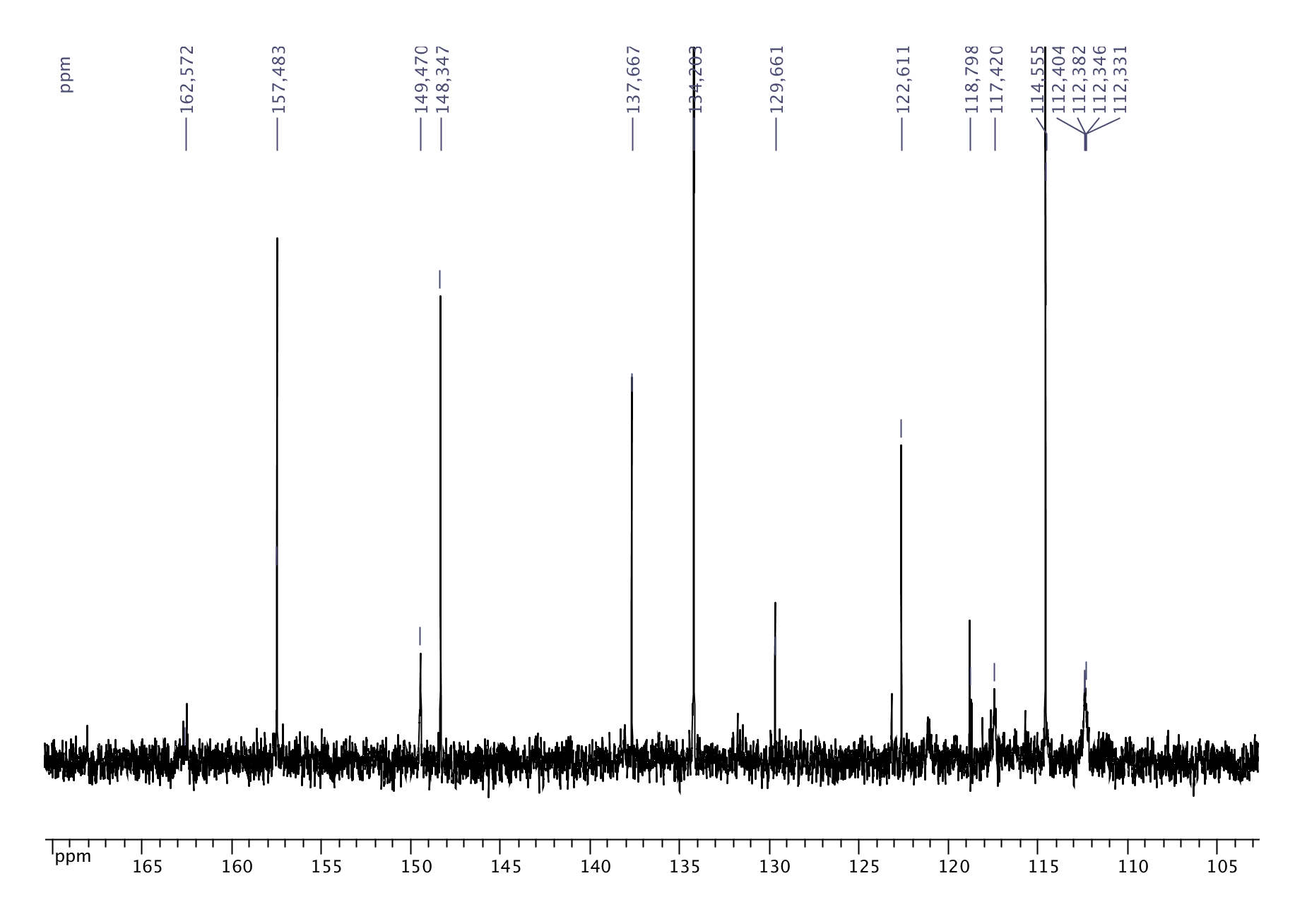


**Figure S7.** ^1^H NMR of compound **5b-2** – CDCl_3_


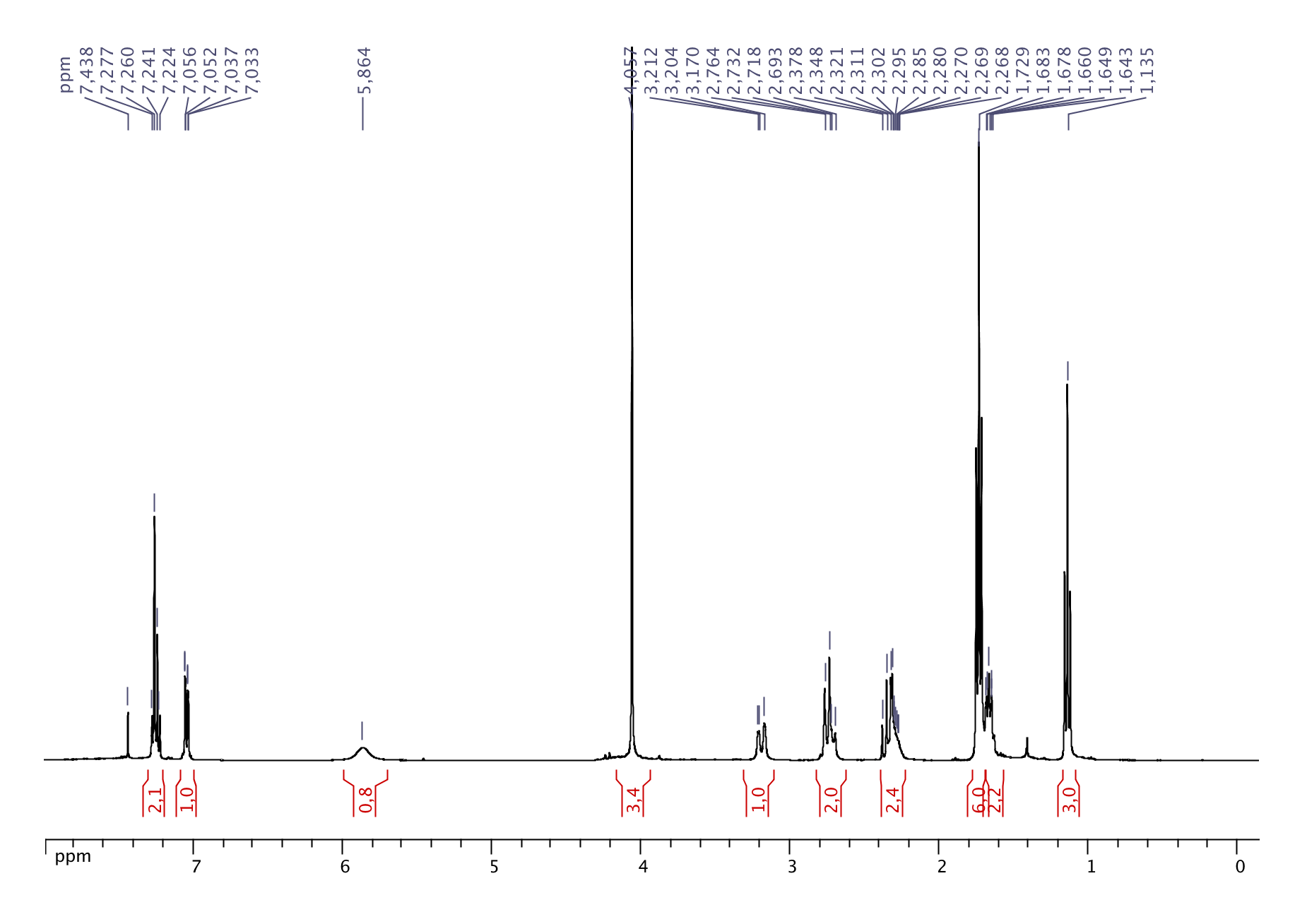


**Figure S8.** ^13^C NMR of compound **5b-2** – CDCl_3_


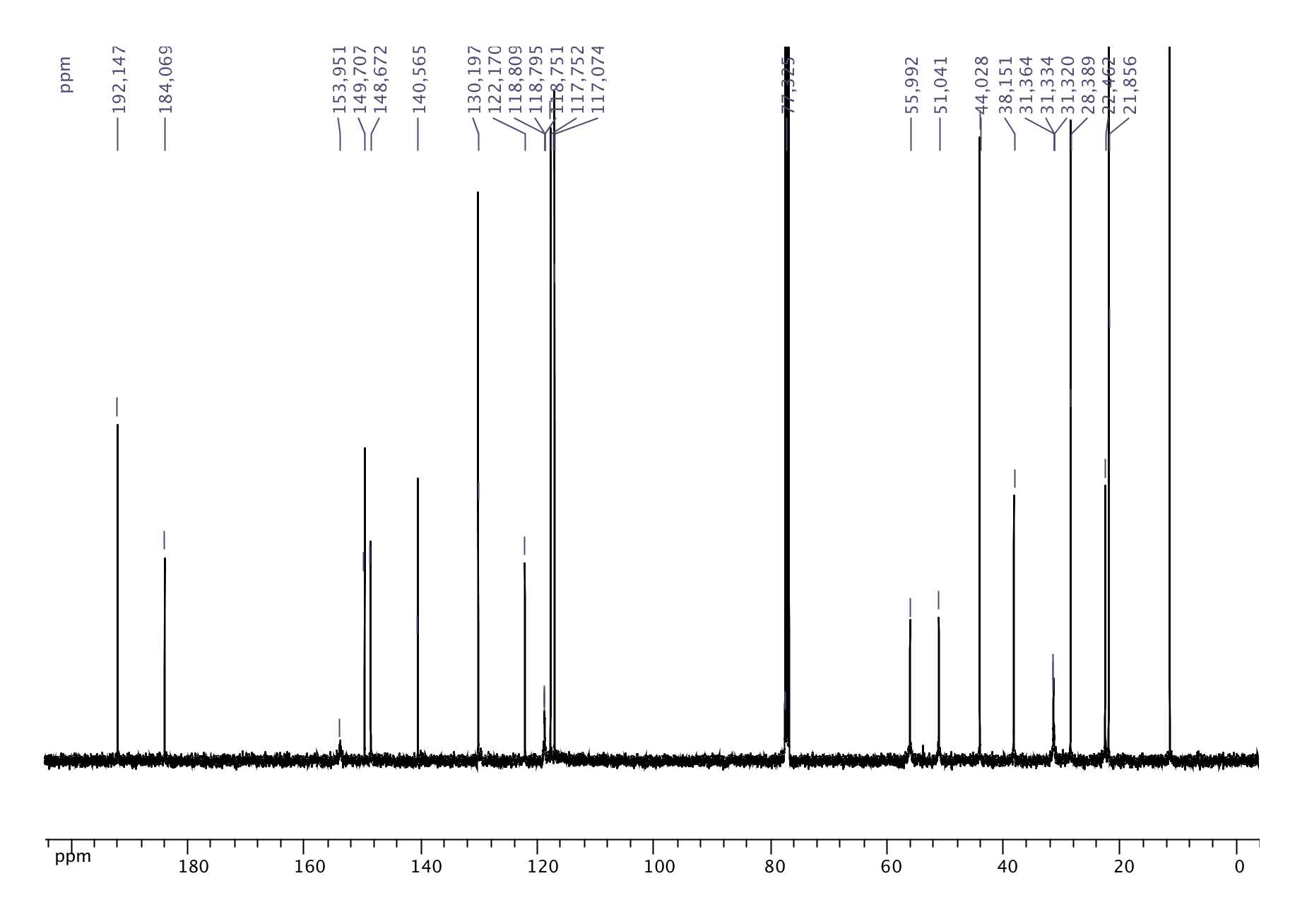


***References***

1. Gozzi GJ, Bouaziz Z, Winter E, Daflon-Yunes N, Aichele D, Nacereddine A, Marminon C, Valdameri G, Zeinyeh W, Bollacke A, Guillon J, Lacoudre A, Pinaud N, Cadena SM, Jose J, Le Borgne M, Di Pietro A. Converting potent indeno[1,2-*b*]indole inhibitors of protein kinase CK2 into selective inhibitors of the breast cancer resistance protein ABCG2. *J Med Chem.* 2015;58:265–277.
2. Bloch S, Nejman-Faleńczyk B, Pierzynowska K, Piotrowska E, Węgrzyn A, Marminon C, Bouaziz Z, Nebois P, Jose J, Le Borgne M, Saso L, Węgrzyn G. Inhibition of Shiga toxin-converting bacteriophage development by novel antioxidant compounds. *J Enzyme Inhib Med Chem.* 2018;33:639-650.
3. Haidar S, Marminon C, Aichele D, Nacereddine A, Zeinyeh W, Bouzina A, Berredjem M, Ettouati L, Bouaziz Z, Le Borgne M, Jose J. QSAR Model of indeno[1,2-*b*]indole derivatives and identification of *N*-isopentyl-2-methyl-4,9-dioxo-4,9-dihydronaphtho[2,3-*b*]furan-3-carboxamide as a potent CK2 inhibitor. *Molecules.* 2019;25:97.
4. Alchab F, Ettouati L, Bouaziz Z, Bollacke A, Delcros JG, Gertzen CGW, Gohlke H, Pinaud N, Marchivie M, Guillon J, Fenet B, Jose J, Borgne ML. Synthesis, biological evaluation and molecular modeling of substituted indeno[1,2-*b*]indoles as inhibitors of human protein kinase CK2. *Pharmaceuticals.* 2015;8:279–302.
5. Alchab F, Fenet B, Le Borgne M, Jose J, Pinaud N, Guillon J, Ettouati L. ^1^H and ^13^C-NMR assignments of bioactive indeno[1,2-*b*]indole-10-one derivatives. *Magn Reson Chem.* 2013;51:837–841.
